# Thinking thrice about sum scores, and then some more about measurement and analysis

**DOI:** 10.3758/s13428-022-01849-w

**Published:** 2022-04-25

**Authors:** Keith F. Widaman, William Revelle

**Affiliations:** 1 School of Education, University of California, 900 University Drive, Riverside, CA 92521 USA; 2 Department of Psychology, Northwestern University, Evanston, IL USA

**Keywords:** Sum scores, Measurement, Reliability, Psychometrics, Latent variable models

## Abstract

Measurement is fundamental to all research in psychology and should be accorded greater scrutiny than typically occurs. Among other claims, McNeish and Wolf (Thinking twice about sum scores. *Behavior Research Methods*, *52*, 2287-2305) argued that use of sum scores (a) implies that a highly constrained latent variable model underlies items comprising a scale, and (b) may misrepresent or bias relations with other criteria. The central claim by McNeish and Wolf that use of sum scores requires the assumption that a parallel test model underlies item responses is incorrect and without psychometric merit. Instead, if a set of items is unidimensional, estimators of reliability are available even if the factor model underlying the set of items does not have a highly constrained form. Thus, dimensionality of a set of items is the key issue, and whether strict constraints on parameter estimates do or do not hold dictate the appropriate way to estimate reliability. McNeish and Wolf also claimed that more precise forms of scoring, such as estimating factor scores, would be preferable to sum scores. We provide analytic bases for reliability estimation and then provide several demonstrations of reliability estimation and the relative advantages of sum scores and factor scores. We contend that several claims by McNeish and Wolf are questionable and that, as a result, multiple recommendations they made and conclusions they drew are incorrect. The upshot is that, once the dimensional structure of a set of items is verified, sum scores often have a solid psychometric basis and therefore are frequently quite adequate for psychological research.

**Supplementary Information:**

The online version contains supplementary material available at 10.3758/s13428-022-01849-w.

## Sum scores and measurement

Measurement is arguably a most basic aspect of our science, as results in all empirical research rest on the measurements we obtain. In our opinion, the field of psychology has paid far too little attention for far too long to important issues in measurement. A case could be made that the current replication crisis roiling psychology and other social sciences cannot be resolved without greater attention to fundamental aspects of the ways we measure characteristics and thereby obtain indicators related to the constructs in our theories (Fried & Flake, 2018).

Recently, McNeish and Wolf (2020) offered a wide-ranging critique of the use of sum scores, arguing against use of sum scores^1^ in favor of estimated factor scores and/or latent variable modeling. The distinction between sum scores and factor scores is, at its basis, a distinction between manifest variable scores and latent variable scores. Manifest variables are those that have scores that “you can get your hands on,” such as the score on a single item, the sum of a set of scale items, a reaction time to a problem presented via computer, or the score on a midterm or final exam in a college course. Latent variables, by contrast, are more esoteric variables, ones that cannot be measured directly. Commonly, latent variables reside as placeholders in complex analytic models, representing theoretical mathematical or statistical entities such as latent factors in exploratory or confirmatory factor models. Ideally, latent variables correspond to the theoretical constructs in our theories, although the degree of alignment of latent variables and associated theoretical constructs is always open to debate. Latent variables cannot be measured directly, so we must use manifest variables to serve as their indicators. Because latent variable scores cannot be calculated directly, they must either be estimated in some fashion or have their distributional properties (e.g., mean, variance) and relations with other variables estimated within the context of complex mathematical models, such as structural equation models.

The past decade has seen a substantial number of reviews of measurement practices in psychology, a refreshing trend given the fundamental importance of measurement. Reporting a reliability coefficient for each scale, although not universal, is becoming more standard (cf. Crutzen & Peters, 2017, for review of articles in health psychology). Revelle and Condon (2019) recommended reporting more than one reliability coefficient for each scale, and various alternative indices of reliability have recently been debated (Bentler, 2021; Cho, 2021; Sijtsma & Pfadt, 2021). But any reliability coefficient is only as good as the psychometric theory on which it is based. One key aspect of psychometric theory is the internal structure of a scale, typically investigated using some form of factor analysis of its items. Unfortunately, this issue is rarely discussed in substantive research studies, as investigators simply sum items in a scale if the scale meets a relatively low bar for adequate reliability (Flake et al., 2017). The factor structure of a set of items is an important form of validity (Borsboom et al., 2004; Messick, 1995) and should impact the ways in which scores are legitimately derived from its items. McNeish and Wolf (2020) summarized this research briefly, arguing that greater attention to various aspects of the psychometric properties of measures is warranted.

McNeish and Wolf (2020) then proceeded with their primary claims: (a) that the use of sum scores is necessarily aligned with a highly constrained latent variable model, often not acknowledged explicitly or implicitly by researchers who use a scale sum score; and (b) that use of sum scores may lead to biased results relative to more precise scores, such as estimated factor scores. We disagree strongly with the first claim, based on more than a century of psychometric work on sum scores, and we feel that the second claim is overblown. In general, we disagree quite strongly that use of sum scores has all of the negative implications and all of the weaknesses McNeish and Wolf describe. To lay out our position, we turn next to what sum scores are and how they relate to reliability and measurement precision.

## Sum scores and reliability

### Sum scores

A sum score, the motivation for the extended disputation by McNeish and Wolf (2020), seems like a very simple operation: for a given scale, compute an equally weighted sum of raw scores of scale items. McNeish and Wolf warned researchers that this seemingly simple and straightforward method may compromise analytic results and, ultimately, the evaluation and interpretation of empirical evidence. This key claim deserves scrutiny.

A general equation for a weighted linear combination or weighted sum score is

1 $$Y={w}_1{X}_1+{w}_2{X}_2+\dots +{w}_p{X}_p$$

where *Y* is the weighted sum of variables *X*_1_ through *X*_*p*_, which can be differentially weighted by the weights *w*_1_ through *w*_*p*_, respectively. If *Y* represents a scale score computed as the sum of *p* items, *X*_1_ through *X*_*p*_, the mean of the weighted combination, $\overline{Y}$, is a weighted combination of the means of the items, ${\overline{X}}_1\ \mathrm{through}\ {\overline{X}}_p$, as

2 $$\overline{Y}={w}_1{\overline{X}}_1+{w}_2{\overline{X}}_2+\dots +{w}_p{\overline{X}}_p$$

In addition, the variance of the weighted sum score, ${\sigma}_Y^2$, is a function of the weighted variances and covariances of the items, written as

3 $${\sigma}_Y^2={w}_1^2{\sigma}_1^2+{w}_2^2{\sigma}_2^2+\dots +{w}_p^2{\sigma}_p^2+2{w}_1{w}_2{r}_{12}{\sigma}_1{\sigma}_2+2{w}_1{w}_3{r}_{13}{\sigma}_1{\sigma}_3+\dots +2{w}_{p-1}{w}_p{r}_{p-1,p}{\sigma}_{p-1}{\sigma}_p$$

where ${\sigma}_j^2$represents the variance of item *j* (*j* = 1, …, *p*), ${r}_{j{j}^{\prime }}{\sigma}_j{\sigma}_{j^{\prime }}$the covariance of items *j* and *j’*, and other terms were defined above.

If unit weighting is used, Eqs. 2 and 3 simplify considerably, as all weights are 1 and hence drop out of the calculations. Then, the scale mean $\overline{Y}$ is simply the sum of the item means, and the variance of the scale ${\sigma}_Y^2$ is the sum of all elements in the matrix of variances on and covariances among the *p* items. Or, if the weights are derived from a factor model, *Y* is said to be an estimate of a person’s standing on a factor (i.e., it is an estimated factor score), but is still simply a linear additive sum of indicators that contain error. The properties of sum scores—the mean and variance—prove valuable in writings on reliability and measurement precision. In early work, Wilks (1938) used these properties to show that a differentially weighted sum score and unit-weighted sum score of the same items have correlations with external variables that become indistinguishable as the number of items increases.

More complex forms of weighting can be used, based on the nature of the variables to be summed. If items differ markedly in variance or if response scales differ across items, one might first standardize each item to *M* = 0, *SD* = 1, prior to summing items. We note that estimation of factor scores, as touted by McNeish and Wolf (2020), is one of an infinite number of forms of differential weighting, albeit a post hoc and sample-specific approach.

McNeish and Wolf (2020) argued that analytic results and associated interpretations of data might be compromised by improper or injudicious use of simple sum scores. But they failed to mention one key strength of sum scores: *If sum scores are computed in exactly the same fashion in different studies, the results of the studies will be more easily compared than if some other, sample-specific method is used in each study to composite item scores.* Of course, this strength holds only if no changes are made across studies to item content or administration. Still, to promote greater replicability of results across studies, sum scores—properly vetted—might have certain benefits over more sophisticated or complex methods of arriving at scores.

Next, we present some basic psychometric ideas, outlining test theory models that have been proposed to evaluate qualities of measures. We then discuss relations between common indices of reliability and their ties to test theory models. In doing so, we argue that sum scores are often sufficiently precise for rigorous research applications, offering a view that contrasts directly with the position propounded by McNeish and Wolf (2020).

### Psychometric basics

Two general families of psychometric models are often contrasted: classical test theory (CTT) and item response theory (IRT). Here, we concentrate on CTT because it is what most applied researchers use in research and is the approach used by McNeish and Wolf (2020). But both technical (e.g., Takane & DeLeeuw, 1987) and expository presentations (e.g., Reise et al., 1993) have discussed the underlying identity of the two approaches. Hence, much of what we have to say from a CTT point of view translates readily to applications using IRT.

#### Reliability as the ratio of true score variance over total variance

One common CTT approach to estimating reliability of scores begins with total test scores (Gulliksen, 1951), where the score of person *i* (*i* = 1, …, *N*) on test *Y* is written as *Y*_*i*_. The test score is assumed to be a function of a person’s performance across a number of items, and the total score on the test for a person is represented as the sum of the person’s true score *T*_*i*_ and an error score *E*_*i*_, as *Y*_*i*_ = *T*_*i*_ + *E*_*i*_. [Note: in the following, we usually dispense with the subscript *i* for person to avoid complicated equations, without loss of generality]. True scores and error scores on a measure are defined as being uncorrelated. As a result, the total score variance of a measure, ${\sigma}_Y^2$, is the sum of true score variance ${\sigma}_T^2$ and error variance ${\sigma}_E^2$, or ${\sigma}_Y^2={\sigma}_T^2+{\sigma}_E^2$. If reliability of test *Y* is represented as *r*_*YY*_ and defined as the ratio of true score variance to total score variance, then

4 $${r}_{YY}=\frac{\sigma_T^2}{\sigma_Y^2}=\frac{\sigma_T^2}{\sigma_T^2+{\sigma}_E^2}$$

where all terms were defined above. This is a most basic formulation of reliability under CTT and underscores the need to devise ways to separate true score variance from error variance.

With this ‘true score plus error score’ formulation for total test scores, several types of reliability can easily be derived. The correlation between scores on a single test administered at two times of measurement is termed a test-retest reliability coefficient. The correlation between scores on two forms of a test, Forms A and B, administered at a single time of measurement is called the parallel forms reliability for each form. Each of these correlations is an estimate of the proportion of reliable or true score variance in scores. Many additional examples could be provided. Importantly, the relatively simple ‘true score plus error score’ model enables a range of interesting ways to portray the reliability of total test scores, computed as sum scores.

#### Item-based approaches

Researchers soon realized that reliability coefficients could be estimated based on a single, multiple-item scale administered at a single point in time. In this approach, items are treated as multiple indicators of the construct assessed. Split-half reliability could be estimated if items were separated into two halves by some rule (e.g., odd- vs. even-numbered) (Brown, 1910; Spearman, 1910). Kuder and Richardson (1937) provided a noted formulation, based on dichotomously scored items. Cronbach (1951) generalized this approach for more quantitatively scored items, leading to the most commonly used item-based coefficient, coefficient alpha, often called Cronbach’s alpha even though Guttman (1945) previously derived the coefficient (see McDonald, 1999). Coefficient alpha, or *α*, can be written as

5 $$\alpha \kern1em =\kern1em \left(\frac{p}{p-1}\right)\left(\frac{\sigma_Y^2-\sum \limits_{j=1}^p{\sigma}_j^2}{\sigma_Y^2}\right)\kern1em =\kern1em \left(\frac{p}{p-1}\right)\left(1\kern0.5em -\kern0.5em \frac{\sum \limits_{j=1}^p{\sigma}_j^2}{\sigma_Y^2}\right)$$

where *p* is the number of items on the scale, ${\sigma}_j^2$ (*j* = 1, …, *p*) is the variance of item *j*, and other terms were defined above. As noted earlier, the variance of a scale, , is equal to the sum of all elements of the matrix of variances and covariances among scale items, with item variances on the diagonal and item covariances off the diagonal. Thus, coefficient *α* is equal to the ratio of the sum of all off-diagonal elements in the item covariance matrix over the sum of all elements, and then multiplied by *p*/(*p* − 1). Cronbach is frequently cited as having shown that coefficient *α* has the attractive quality of being the average of all possible split-half reliability coefficients.

Coefficient *α* is often called an internal consistency reliability coefficient, as it is based on covariances among scale items and thus internal consistency among items. But internal consistency should not be conflated with homogeneity, where homogeneity implies that a single dimension underlies the set of items. Coefficient *α* is *not* a homogeneity coefficient, as *α* can be relatively high even if multiple dimensions are present in a scale (Green et al., 1977; Green & Yang, 2009; Revelle, 1979; Revelle & Wilt, 2013; Revelle & Zinbarg, 2009; Schmitt, 1996; Zinbarg et al., 2006). Recognition of this issue led to the development of factor analytically based representations of scales, discussed next.

### Psychometric models and their factor analytic analogs

As McNeish and Wolf (2020) noted, items comprising a scale should be factor analyzed to determine if the scale is best represented by only a single dimension. If a single factor well represents covariances among items, the scale would be considered a homogeneous scale, and a single score of some sort across all scale items is quite reasonable. Or, if two or more factors are required to represent relations among a set of items, then use of a single score may misrepresent what the scale assesses and distort or mask important features of the data (see Bentler, 2021).

Under CTT, researchers distinguished several types of psychometric models, shown in Table 1. Parallel tests were defined as two or more tests that have (a) equal means, (b) equal true score variances, (c) equal error variances, and (d) equal correlations with external variables, a rather daunting set of requirements. Because of characteristics (b) and (c), parallel tests also have equal reliabilities. Converting these conditions to a factor analytic representation, consider a set of three indicators that are hypothesized to reflect a single latent factor. The linear model for these indicators could be written, closely mimicking a set of regression models, as

6 $${\displaystyle \begin{array}{l}{X}_1={\nu}_1+{\lambda}_1T+{\varepsilon}_1\\ {}{X}_2={\nu}_2+{\lambda}_2T+{\varepsilon}_2\\ {}{X}_3={\nu}_3+{\lambda}_3T+{\varepsilon}_3\end{array}}$$

where *X*_1_ through *X*_3_ are scores for an individual on the three manifest indicators (e.g., scale items), *ν*_1_ through *ν*_3_ are intercepts for manifest indicators, respectively, *λ*_1_ through *λ*_3_ are loadings of the three indicators on latent variable *T*, or, effectively, regression coefficients for predicting manifest variable scores from the latent variable *T*, and *ε*_1_ through *ε*_3_ represent errors of measurement. In this model, each person has one score on each of the manifest variables *X*_1_ through *X*_3_, has a score on the single common factor *T* underlying the set of manifest variables, and has an associated error of measurement *ε*_1_ through *ε*_3_ for each equation, respectively. If we define true score *T* to have *M* = 0, and *SD* = 1, each measurement error variate to have *M* = 0, and positive *SD*, and measurement errors to be mutually uncorrelated and uncorrelated with the latent factor *T*, the characteristics of a set of parallel measures can be easily presented. Because true score *T* and error scores have means of zero, the intercepts in Eq. 6 become the means of the manifest variables. Because the variance of *T* is 1.0, the square of each *λ* provides the raw-score estimate of true-score variance in an indicator, and the variance of each error term reflects error variance.^2^ Modern structural equation modeling software makes constraining parameter estimates to equality a simple matter. Consider a factor model with the following constraints: The three intercepts/means *ν*_1_ through *ν*_3_ are constrained to equality, the three loadings *λ*_1_ through *λ*_3_ are constrained to equality, and the variances of the three error terms *ε*_1_ through *ε*_3_ are constrained to equality. If such a model fits a data set well, the model would satisfy three of the key requirements of a *strictly parallel test* model for the data (cf. McDonald, 1999), notably equal means, equal true score variances, and equal error variances. If means of variables are not considered or are allowed to vary, even though factor loadings are constrained to equality as are the error variances, such a model can be characterized as a model for *essentially parallel tests*.

**Table 1:** Psychometric characteristics of different types of tests

Test type	Means	True Score Variance	Error Variance	Correlations w/ Other Variables
Strictly parallel tests	Equal	Equal	Equal	Equal
Essentially parallel tests	--	Equal	Equal	Equal
Strictly tau equivalent	Equal	Equal	Vary	Vary
Essentially tau equivalent	--	Equal	Vary	Vary
Congeneric	--	Vary	Vary	Vary

If a model with full parallel test constraints does not have acceptable fit to data, less constrained statistical models might be considered. For example, if the means *ν*_1_ through *ν*_3_ are constrained to equality and the loadings *λ*_1_ through *λ*_3_ are constrained to equality, but variances of measurement error terms *ε*_1_ through *ε*_3_ are allowed to vary, this may be termed a *strictly tau equivalent test* model. The term *tau equivalent* is used because *tau* is the Greek name for *T*, the true score, and a strictly tau equivalent model retains equality of linear relations of the true score to each manifest variable, even as the variances of errors of measurement are allowed to vary across indicators. Although tau equivalent indicators have equal true score variances, they have different reliabilities based on their different error variances. As with parallel tests, if the means of tau equivalent tests are either not part of the model or are allowed to vary across tests, the resulting model is identified as an *essentially tau equivalent test* model.

As a final step, if all parameter estimates in the model—means (i.e., intercepts), factor loadings, and error variances—are allowed to vary across indicators, but the set of indicators is well represented by a single latent factor, the resulting model is termed a *congeneric test* model. The congeneric test model is clearly the least constrained of the test theory models. But if the single-factor congeneric test model provides adequate fit to a set of indicators, the indicators can be considered a homogeneous set of measures, and a single score derived from the measures is theoretically and mathematically justified and interpretable.

Our reason for emphasizing psychometric models is this: the models have ties to indexes of reliability. As experts have shown (e.g., McDonald, 1970, 1999; Revelle & Zinbarg, 2009), coefficient *α* will yield an accurate model-based estimate of reliability only if scale items conform at least to an essentially tau equivalent structure, such that items have equal factor loadings in addition to loading on a single factor. A concise set of requirements is (a) the set of items is unidimensional (i.e., a single factor underlies covariances among items), (b) measurement error terms do not covary (i.e., no residual covariances among residual terms are present), (c) items are linearly related to the factor, and (d) the set of items has at least essential tau equivalent structure (i.e., equal factor loadings). If these four characteristics hold, then coefficient *α* yields an estimate of reliability of the equally weighted sum of items that is equal to the model-based coefficient omega, which is discussed immediately below.

If the first three of the preceding characteristics (a) through (c) hold, but the last one does not—that is, factor loadings vary across items, then coefficient *α* is not a recommended estimator of scale reliability. In this case, McDonald (1970, 1999) derived an alternative estimator, coefficient *ω*_*T*_,^3^ for congeneric test models with a single factor that can be calculated as:

7 $${\omega}_T=\frac{{\left(\sum \limits_{j=1}^p{\lambda}_j\right)}^2}{{\left(\sum \limits_{j=1}^p{\lambda}_j\right)}^2+\sum \limits_{j=1}^p{\theta}_j^2}$$

where ${\theta}_j^2({j}=1, . . .,p)$ is the variance of measurement error ${e}_j$, and all other symbols were defined above. Thus, to compute coefficient *ω*_*T*_, one first sums the loadings of the *p* items and squares this sum, placing the result in the numerator, as this represents the total amount of true score variance across the set of items. The denominator consists of the sum of the true score variance plus the sum of the measurement error variances of the *p* items. Equation 7 therefore is one instantiation of Eq. 4, that a reliability coefficient should be the ratio of true score variance over total scale variance, in this case a ratio based on a congeneric test structure.

Derivations of reliability coefficients such as coefficients *α* and *ω*_*T*_  are based on raw scores for items, not standardized item responses. If raw item scores are used to compute sum scores, as is common practice, then only raw score, or covariance metric, parameter estimates should be used in equations such as Eqs. 5 and 7 to estimate scale reliability.

Another issue of considerable importance is the fact that most prior derivations of coefficients alpha and omega and relations between them have considered only population values of parameters, such as those in Eqs. 5, 6, and 7, and this limits certain generalizations that may be drawn. In any sample, sample-based estimates of variances (e.g., in Eq. 5) or of factor loadings and unique variances (e.g., in Eqs. 6 and 7) take the place of population parameters in those equations. All claims about alpha and omega hold only for strictly unidimensional data structures, such that a single factor accounts perfectly for all covariances among items. In such data structures, coefficients alpha (from Eq. 5) and omega (from Eq. 7) will attain identical values if loadings of items on the single factor are equal; if item loadings on the factor are unequal, alpha will lead to a lower value than omega, although the difference is often small in magnitude. Strictly unidimensional data structures probably hold only in population structures “… in the bowels of a computer processor running a Monte Carlo study” (borrowing a phrase from Cohen, 1990), structures that exhibit no misfit in the population. Even there, when simulating sample data from such populations, a single factor will fail to fit item covariances perfectly in a given sample due to sampling error (MacCallum & Tucker, 1991), leading to some qualifications of alpha vs. omega claims. Furthermore, most empirical data are not strictly unidimensional, but are at best only approximately or essentially unidimensional, so have additional small perturbations of item covariances that preclude perfect fit of a one-factor model even in the population (e.g., minor domain factors too numerous to model, as posited by Tucker et al., 1969; see also Nguyen & Waller, 2022). Calculation of coefficient alpha using Eq. 5 treats all off-diagonal covariation among items as true-score-related variance. In contrast, calculation of coefficient omega using Eq. 7 treats only the off-diagonal item covariation accounted for by the single factor as true-score-related variance. If the single factor fails to explain all off-diagonal item covariance, it is theoretically possible for coefficient alpha to attain a higher value than coefficient omega. Although not highlighted in their paper, Deng and Chan (2017), using empirical sample data, reported a small number of instances in which coefficient alpha led to a higher estimate of reliability than did coefficient omega, almost surely due to the lack of strict unidimensionality of scale items in these instances.

### Sum scores and factor models

McNeish and Wolf (2020) noted that an equally weighted sum of raw scores of manifest variables can be represented as a parallel test model with constrained equal factor loadings and constrained equal error variances. McNeish and Wolf proceeded to claim that a researcher who uses an equally weighted sum of items assumes implicitly that a single-factor parallel test model well represents the relations among the set of items, an assumption that should be acknowledged explicitly and tested. This claim is fundamentally flawed.

The fundamental flaw is this: employing an equally weighted sum of a set of items does not imply that a parallel test model is the proper representation of covariances among items, as if the parallel test model was the justification for computing the sum score. Instead, the implication flows in the opposite direction: if a one-factor model of *any* form provides close fit to covariances among items, an equally weighted sum of items is a reasonable basis for estimating reliability, but the form of the factor model dictates how reliability should be estimated.

The basis for these assertions rests on Eqs. 3, 5, and 7. Using unit weights in the summation, Eq. 3 shows that the variance of the sum of a set of items is the sum of all elements of the matrix of covariances among items. If this covariance matrix is well explained by a one-factor model, Eq. 5 can be used if all factor loadings on the common factor are equal, because such a model is consistent with equal off-diagonal values.^4^ If factor loadings vary, then Eq. 7 should be used to obtain an estimate of reliability. Thus, good fit of a one-factor model justifies use of an equally weighted sum of items, and the nature of the one-factor model dictates the formula to use when estimating reliability of the sum score.

We note that all of the foregoing relies on test theory approaches based on the assumption of strict homogeneity across a set of items, which implies the presence of a single dimension underlying the items. If a set of indicators is multidimensional, an equally weighted sum of the items may still be justified, but this relies on altered test theory approaches. Measures often have a bifactor structure, with one dominant, general factor on which all items load, and then a set of orthogonal group factors on which subsets of items load. Variants of Eq. 7 can be used in such situations, which led Zinbarg et al. (2005) to distinguish coefficient *ω*_*H *_(which estimates reliability associated only with the general factor) from coefficient *ω*_*T *_(which estimates reliability across the total set of general and all group factors) (see, e.g., Revelle & Zinbarg, 2009; Revelle & Condon, 2019; Zinbarg et al., 2006).

Alternatively, one might design measures based on the theory of parallel forms. If one intended to assess general intelligence, a wide-ranging, heterogeneous battery of indicators might be desired. The battery would assess a bit of this, a bit of that, and a bit of other things, all within the general domain of mental ability. Such a battery might be quite multidimensional, but a single score across the entire battery could have substantial levels of reliability (e.g., parallel forms reliability with another battery with similar content specification) (cf. Cronbach, 1951). Extended consideration of multidimensional collections of indicators is beyond the scope of this paper. However, we mentioned this issue here to emphasize that unidimensionality and homogeneity are not the only bases available for developing reliability theory.

### Factor analytic population parameters and sample estimates

Yet another matter that deserves attention is the issue of factor analytic population parameters and their sample estimators, an issue that McNeish and Wolf (2020) failed to discuss. Factor scores are subject to several forms of indeterminacy. One form of factor score indeterminacy is mathematical in nature, because the number of common plus unique factors is greater than the number of manifest variables analyzed. Hence, factor scores can only be estimated, not calculated in any deterministic sense. Second, even in the population, multiple methods of factor score estimation are available, and the methods yield different estimated factor scores with different properties (see, e.g., Grice, 2001; Tucker, 1971). Third, in any sample, factor loadings, which are used in sample-based estimation of factor scores, will vary from sample to sample and from their population values, leading to fluctuations in factor scoring weights and thus additional indeterminacy from sample to sample and from sample to population (MacCallum & Tucker, 1991). Fourth, manifest variables are standardized in a sample to a mean of zero (and typically also a *SD* = 1.0) prior to being weighted and summed in the estimation of factor scores. As a result, estimated factor scores have a mean of zero in any sample. Because manifest variable means (and *SD*s) vary from sample to sample and from sample to population, this is yet another sample-specific form of indeterminacy built into the estimation of factor scores.

Sum scores, using unit weights, circumvent certain aspects of indeterminacy through the use of identical weights in every sample, so the weights are not subject to sample-by-sample fluctuation. Furthermore, whether to transform manifest variables prior to summing them is a matter to be scrutinized, rather than dictated as in sample-based standardization used in estimating factor scores. If manifest variables are on approximately the same scale (e.g., a set of items each answered on a 1-to-7 scale), researchers typically do not transform scores, but simply sum the raw scores on the variables.^5^

Sum scores are fallible (i.e., imperfect) estimates of relative standing of individuals on the dimension implied by the sum score and purport to be nothing more. But, in most respects, estimated factor scores can be described in similar fashion. Both sum scores and estimated factor scores are weighted sums of scores on manifest variables, so have less-than-perfect reliability. Sum scores are equally weighted sums of manifest variable scores that are usually summed in their raw score form; estimated factor scores are differentially weighted sums of the same manifest variables, using sample-specific weights applied to scores subjected to sample-specific standardization. McNeish and Wolf (2020) clearly preferred estimated factor scores over sum scores. But whether the various forms of sample specificity involved in the estimation of factor scores lead to scores with noticeably superior properties relative to sum scores is a matter for empirical investigation, not a priori decree.

## Estimating reliability in empirical data

### Holzinger-Swineford (Holzinger & Swineford, 1939) data

To serve as the empirical basis for their demonstrations, McNeish and Wolf (2020) introduced a set of six manifest variables derived from the classic study by Holzinger and Swineford (1939). McNeish and Wolf referred to the variables as “six items from a cognitive ability assessment …,” implied they were items for a single cognitive dimension, and stated that item scores varied from 0 to 10. These claims are incorrect in at least three important ways. First, the six “items” were not single items, but were six tests of cognitive ability, with scores on each test being a sum score across many items. Second, the variables were not developed to be indicators for a single dimension. Instead, the first three variables—paragraph comprehension, sentence completion, and word meaning—were indicators for a Verbal factor, and the last three variables—addition, counting dots, and straight-curved capitals—were designed to be indicators for a Speed factor. Third, score ranges were not strictly comparable across tests, as McNeish and Wolf (2020) stated. The original raw scores on the six variables were quite different across variables in terms of their *M*s, *SD*s, and score ranges, and the *scaled* scores for these variables (linearly rescaled versions of the raw scores available through the lavaan (Rosseel, 2012, 2021) and psychTools (Revelle, 2021b) packages in R) have more similar means but score ranges that vary across tests. Finally, in a paper with a goal of promoting precision in measurement and analyses, we are unsure why McNeish and Wolf decided to degrade information in the scaled scores by rounding the scores to integer values. Rounding of scores led to less information in the rounded scores, as decimal places in the scaled scores provided useful individual difference information. Additional details on the nature of the six tests and the various ways in which they were scored and transformed are provided in Supplemental Material.^6^

In our analyses reported below, we used the more precise scaled scores, and we have provided analytic results using the rounded scores in Supplemental Material for completeness and comparison with our results. As shown in Table 2, the skewness and kurtosis values for the six tests are not extreme, implying that each of the six variables was approximately normally distributed. In addition, as hypothesized by Holzinger and Swineford (1939), the three verbal tests (x06, x07, and x09) were relatively highly correlated, the three speed tests (x10, x12, and x13) were also relatively highly correlated, and the three speed tests correlated at relatively low levels with the three verbal tests (see Table 2).

**Table 2:** Descriptive statistics and correlations for scaled test scores from Holzinger and Swineford (1939)

Test	Description	*M*	*SD*	Skew	Kurt	Min	Max
Descriptive statistics							
x06	Paragraph comprehension	3.06	1.16	0.27	0.08	0.00	6.33
x07	Sentence completion	4.34	1.29	-0.35	-0.55	1.00	7.00
x09	Word meaning	2.19	1.10	0.86	0.82	0.14	6.14
x10	Addition	4.19	1.09	0.25	-0.31	1.30	7.43
x12	Counting dots	5.53	1.01	0.53	1.17	3.05	10.00
x13	Straight-curved capitals	5.37	1.01	0.20	0.29	2.78	9.25
Correlations		x06	x07	x09	x10	x12	x13
x06	Paragraph comprehension	1.00					
x07	Sentence completion	.73	1.00				
x09	Word meaning	.70	.72	1.00			
x10	Addition	.17	.10	.12	1.00		
x12	Counting dots	.11	.14	.15	.49	1.00	
x13	Straight-curved capitals	.21	.23	.21	.34	.45	1.00

Elements in the Test column identify manifest variables, where “x”’ stands for the scaled (or transformed) versions as provided by the lavaan program, and the numbers (06, 07, etc.) refer to the ordinal position of tests in the Holzinger and Swineford (1939) protocol and monograph.

### Factor scores versus sum scores

McNeish and Wolf (2020) devoted considerable attention to the contrast between estimated factor scores from a one-factor congeneric test model fit to the six manifest variables with the simple sum score across the six variables. They emphasized that estimated factor scores from the congeneric test model correlated at the rather low level of *r* = .87 with sum scores. McNeish and Wolf highlighted the rather different estimated factor scores for two particular individuals, even though the individuals had identical sum scores across the six tests. Thus, estimated factor scores suggested the two individuals differed substantially in level of cognitive ability, whereas sum scores implied the individuals had equal levels of ability. Unfortunately, this section of the McNeish and Wolf paper is based on theoretical and empirical mistakes and misapprehensions.

One theoretical misapprehension is the presumption that estimated factor scores are the best scores to be obtained from a set of indicators, decidedly better than sum scores. If factor scores reveal large differences between certain persons who have identical sum scores, the factor scores were presumed to be more optimal and sum scores suspect. But, given variation in factor loadings and their factor scoring weights across samples, estimated factor scores in one sample will be based on one set of scoring weights in one sample and a different set of scoring weights in other samples. As a result, comparisons across samples of relations of factor scores with other variables will have a bit of an “apples versus oranges” flavor. In contrast, although sum scores may not be ideal in all ways, the use of the same, unit compositing weights across samples helps ensure that apples are compared to apples, even if they have some blemishes.

A second issue involves a bit of sleight of hand by McNeish and Wolf (2020), who branded the different characterizations of individuals as a demonstration that seemingly more precise estimated factor scores yield more useful and insightful information about the relative standing of individuals on an underlying dimension than do stodgy sum scores. The sleight of hand is this: earlier in their paper, McNeish and Wolf had argued that estimated factor scores from a parallel test model correlate perfectly (i.e., *r* = 1.0) with simple sum scores, so sum scores represent (implicitly) the estimated factor scores from a parallel test model. Thus, rather than a “contest between estimated factor scores versus sum scores” (to the detriment of sum scores), one could characterize their discussion, in all relevant respects, as a “contest between estimated factor scores from a congeneric factor model versus estimated factor scores from a parallel test model.” That is, both sets of scores are the seemingly more precise way to score manifest variables (i.e., both are estimated factor scores, according to McNeish and Wolf), but the two sets of factor scores yield very different information at the individual level. The key issue becomes the relative fit and interpretability of alternative factor models, and sum scores have only a tangential relation to this contrast.

This issue of the fit of different one-factor models highlights a signal empirical mistake by McNeish and Wolf (2020)—they failed to follow their own advice and the advice of other recent commentators to base scores on the internal structure of the six manifest variables. If they had, McNeish and Wolf would have had to confront several troubling issues. At various points, McNeish and Wolf reported fitting one-factor parallel test and one-factor congeneric test models to the six variables and also fit a two-factor congeneric model to the data. For completeness, we fit six models to the data—one-factor parallel, tau equivalent, and congeneric test models, and two-factor versions of each model. Fit indices for these models are shown in Table 3. To evaluate fit, we used the standard *χ*^2^ test of model misfit, whereby a significant *χ*^2^ value provides a statistical basis for rejecting a model. The *χ*^2^ value is directly related to sample size, so indices of practical fit are often recommended. We used several practical fit indices. One was the root mean square error of approximation (RMSEA) with its 90% confidence interval (CI). Values of RMSEA of .05 or lower index close fit of a model to data, .05 to .08 adequate fit, .08 to .10 poor fit, and above .10 unacceptable fit. The comparative fit index (CFI) and Tucker–Lewis index (TLI) are measures of off-diagonal covariation explained by a model, and values of .95 or higher reflect close model fit. The standardized root mean square residual correlation (SRMR) is a measure of the average residual correlation, and values below .08 are desirable. The preceding criteria for close model fit are not hard-and-fast cutoffs, but should be considered approximate indicators of close fit, and their use is buttressed by simulation results (e.g., Hu & Bentler, 1999).

**Table 3:** Fit of six alternative factor models to scaled scores (x06–x13)

Model	No. of		Statistical fit			Practical fit			
number	factors	Psychometric model	χ^2^	*df*	Prob	RMSEA [CI]	CFI	TLI	SRMR
1	1	Essentially parallel	398.72	19	<.0001	.258 [.236, .280]	.430	.550	.254/.195
2	1	Essentially tau equivalent	383.48	14	<.0001	.296 [.271, .322]	.446	.406	.251/.198
3	1	Congeneric	149.79	9	<.0001	.228 [.197, .261]	.789	.648	.130/.130
4	2	Essentially parallel	40.62	16	.0006	.072 [.045, .099]	.963	.965	.104/.063
5	2	Essentially tau equivalent	29.10	12	.004	.069 [.037, .101]	.974	.968	.071/.049
6	2	Congeneric	14.35	8	.073	.051 [.000, .093]	.990	.982	.034/.034

RMSEA [CI] = root mean square error of approximation and its 90% confidence interval, CFI = comparative fit index, TLI = Tucker–Lewis index, SRMR = standardized root mean square residual. ^a^ In the SRMR column, the value before the slash is from output based on analysis with Mplus, and the value after the slash is from output after analysis using lavaan package in R.

Inspection of fit indices in Table 3 will reveal that Models 1, 2, and 3—the one-factor parallel, tau equivalent, and congeneric models—had wholly inadequate fit, having rejectable statistical fit and extremely poor practical fit. Thus, the entire presentation by McNeish and Wolf (2020) of the differences between congeneric factor scores and sum scores—favoring estimated factor scores over sum scores—is moot. The estimated factor scores from a one-factor congeneric test model and the sum scores across all six tests (which correlate perfectly with parallel test estimated factor scores) have no empirical justification or valid interpretation.

The fit indices for Models 4, 5, and 6—the two-factor parallel, tau equivalent, and congeneric models—have much better fit. We consider the parallel and tau equivalent models to be improper when fit to the six tests, because models with factor loadings constrained to equality should be entertained only if indicators were carefully designed to have equal or approximately equal measurement scales and, thus, variances. Test descriptions provided by Holzinger and Swineford (1939) and descriptive statistics for raw scores shown in Supplemental Material Table S1 demonstrate this was not the case for these six variables. The transformations resulting in scaled scores led to more similar means and *SD*s across tests, but the transformations were never explicitly justified. An infinite number of alternative, arbitrary, linear transformations are allowable, and these would affect measures of the fit of factor models with constraints on loadings, but would leave the fit of congeneric factor models unchanged. Thus, only congeneric factor models are psychometrically legitimate contenders for the six HS data variables, and the two-factor congeneric model had very close fit to the data, as shown in Table 3.

In Table 4, parameter estimates from the two congeneric factor models are shown. In the one-factor model, the three verbal tests had rather high loadings (0.91 and higher), and the three speed tests had very low loadings (0.28 or lower). This pattern of loadings implies that the factor was very closely aligned with verbal ability, and thus accounted for very little variance in speed variables. Not surprisingly, error variances for the three verbal tests were relatively small, and error variances for the speed tests were extremely large. In the two-factor model, loadings on the Verbal factor and error variance estimates for the three verbal tests were quite similar to those in the one-factor model. But loadings on the Speed factor and error variance estimates for the three speed variables were dramatically altered, with much higher loadings and much reduced error variances, as the two-factor model was able to represent well the speed variables with the additional latent variable. The correlation between the two factors, *r* = .26, indicates that the two factors were not highly correlated. The very good fit indices for the two-factor congeneric model indicate that it is the optimal model for the six scaled variables.

**Table 4:** Raw score parameter estimates for two congeneric CFA models fit to scaled scores

		One-factor model		Two-factor model		
				Loading		
Variable	Test Description	Loading	Error variance	Verbal	Speed	Error variance
x06	Paragraph comprehension	0.98	0.38	0.98	0*	0.38
x07	Sentence completion	1.11	0.42	1.11	0*	0.42
x09	Word meaning	0.91	0.37	0.91	0*	0.37
x10	Addition	0.19	1.15	0*	0.67	0.73
x12	Counting dots	0.18	0.99	0*	0.78	0.42
x13	Straight-curved capitals	0.28	0.94	0*	0.59	0.67
Factor 1		1.0*		1.0*		
Factor 2				0.26	1.0*	

Tabled values in the top section of the table are estimated factor loadings and error variances (or unique factor variances); in the bottom section, factor variances and covariances are reported. Asterisked parameters were fixed to reported values to identify the solution.

### Demonstration #1: reliability estimates

The six scaled test scores derived from the Holzinger and Swineford (1939) study are quite useful for demonstrating various ways of estimating reliability in empirical studies.

#### Alpha and omega

Because the two-factor congeneric test model shown in Table 4 is a reasonable representation of data, we can contrast coefficient *α* and *ω*_*T *_values separately for the three verbal tests and the three speed tests. In the top half of Table 5, the covariances among the three verbal tests are shown, along with the factor loadings and error (or unique) variances from a one-factor congeneric model fit to the (3 × 3) covariance matrix. Equation 5 illustrates that the sum of all elements of the covariance matrix and the sum of the diagonal values are needed to calculate coefficient *α*, and Eq. 7 shows that the sum of factor loadings and the sum of unique variances are required to calculate coefficient *ω*_*T*_. Simple calculations show that *α* = .883 and *ω*_*T*_ = .886, very similar values given the similarity of loadings on the Verbal factor.

**Table 5:** Coefficients α and *ω*_*T*_ for verbal and speed sum scores based on scaled scores from Holzinger and Swineford (1939)

Variable names	Covariance matrix			Factor model		Reliability	
Verbal tests	x06	x07	x09	*λ*	*θ*	*α*	*ω*_*T*_
x06 Paragraph Compr	1.355	1.101	0.899	.984	.382		
x07 Sentence Compl	1.101	1.665	1.018	1.115	.416		
x09 Word meaning	0.899	1.018	1.200	.910	.369	.883	.886
Speed tests	x10	x12	x13	*λ*	*θ*	*α*	*ω*_*T*_
x10 Addition	1.187	0.537	0.375	.661	.746		
x12 Counting Dots	0.537	1.025	0.459	.801	.366		
x13 S-C Caps	0.375	0.459	1.018	.565	.696	.689	.696

For variable names, Compr = comprehension, Compl = completion, S-C Caps = straight-curved capitals. In the Factor model columns, λ = factor loading, and θ = error/unique variance.

In the bottom half of Table 5, comparable values for speed tests are shown, with the (3 × 3) covariance matrix and factor loadings and unique variances from a congeneric model fit to the covariance matrix. For speed tests, reliability coefficients were noticeably lower, with *α* = .689 and *ω*_*T*_ = .696, but still rather similar values despite apparent variability in loadings on the Speed factor. The upshot of these analyses is that a verbal sum score had relatively high levels of homogeneity reliability, and a speed sum score had lower and borderline levels of reliability.

#### Correlations among estimated and true factor scores and sum scores

Recently, Nicewander (2020) proposed ways of estimating the correlation of estimated factor scores with true factor scores and extended this to estimating the correlations of sum scores with true factor scores and with factor score estimates. Because reliability is the proportion of variance in a score associated with a true score, the squares of correlations of factor score estimates and sum scores with true factor scores yield estimates of reliability. Notably, all of these correlations can be estimated from factor analytic results without the actual estimation of factor or sum scores.

Restricting attention to single-factored applications, let **C**_*xx *_stand for the (*p* × *p*) matrix of covariances among *p* manifest variables, a superscript (-1) for the inverse operation, **λ **for the (*p* × 1) column vector of factor loadings, **x **for the (*p* × 1) column vector of scores on *p* manifest variables, $\hat{f}\ \mathrm{and}\ f$ for estimated and true factor scores, respectively, and **1** for the (*p* × 1) column vector of ones (to be used for summing, such that **1**^′^**x **is the sum of manifest variable scores for an individual). Nicewander (2020) showed that *ρ*, the population correlation of regression estimate factor scores with true factor scores, can be represented as

8 $$\rho \left(\hat{f},f\right)={\left[{\boldsymbol{\uplambda}}^{\prime }{\mathbf{C}}_{xx}^{-1}\boldsymbol{\uplambda} \right]}^{1/2}$$

and the square of this value

9 $${\rho}^2\left(\hat{f},f\right)={\boldsymbol{\uplambda}}^{\prime }{\mathbf{C}}_{xx}^{-1}\boldsymbol{\uplambda}$$

is the reliability of the estimated factor scores. Similarly, the squared correlation between the sum score and true factor scores, and thus reliability of sum scores, can be written as

10 $${\rho}^2 \left({\mathbf{1}}^\prime{\mathbf{x}},f\right)={\mathbf {\left({1^\prime}{\uplambda} \right)}^2}/[\mathbf{1^\prime{C}}_{xx}{\mathbf{1}}]$$

and the correlation of estimated factor scores with sum scores can be written as

11 $$\rho \left(\hat{f},{\mathbf{1}}^{\prime}\mathbf{x}\right)=\left({\mathbf{1}}^{\prime}\boldsymbol{\uplambda} \right)/\left[{\left({\boldsymbol{\uplambda}}^{\prime }{\mathbf{C}}_{xx}^{-1}\boldsymbol{\uplambda} \right)}^{1/2}{\left({\mathbf{1}}^{\prime }{\mathbf{C}}_{xx}^{-1}\mathbf{1}\right)}^{1/2}\right]$$

where symbols were defined above. Replacing all vectors and matrices in Eqs. 8 through 11 with their sample estimates leads to sample estimates of associated values. Additional details and programs for estimating these values are presented in Supplemental Material.

Based on the above, estimated factor scores on the Verbal factor have a reliability of .886, sum scores a reliability of .886, and sum scores and estimated factor scores correlate very highly, *r* = .9999. Results for the Speed factor are noticeably lower. Estimated factor scores on the Speed factor have a reliability of .739, sum scores a reliability of .696, and sum scores and estimated factor scores correlate fairly highly, *r* = .970. Thus, reliabilities for estimated factor scores using the Nicewander (2020) approach tend to be slightly or moderately higher than coefficient *ω*_*T*_ reliabilities for sum scores.

#### Composite reliability

If manifest variables are single items or are test scores for which reliability estimates are unavailable, the preceding methods are perhaps the only usable way to estimate reliability. However, still another alternative is possible. Cronbach (1951) outlined how to estimate reliability of what he called a *lumpy* test. In a similar vein, Rae (2007) provided a readable account of estimating reliability of a composite that is formed as the sum of several components, if each component is accompanied by an estimate of reliability. Fortunately, Holzinger and Swineford (1939) reported reliability coefficients for each test in their battery.

The composite reliability method from Rae (2007) is conceptually simple to calculate. The sum of all elements of the matrix of raw covariances among manifest variables is the total variance of the composite. Given an estimate of reliability of each manifest variable, what we term a *reduced* covariance matrix is identical to the raw covariance matrix except that each diagonal value is replaced by the variance of the variable multiplied by its reliability. Thus, the diagonal values of this matrix contain an estimate of true score variance in each variable, and the sum of all values in the reduced covariance matrix yields an estimate of true score variance in the composite. The ratio of true score variance of the composite over the total score variance of the composite is therefore an estimate of the reliability of the composite, or composite reliability.

Calculations for composite reliability are shown in Table 6. In the top half of Table 6, results for the verbal tests are shown, specifically, the raw covariance matrix, the reliability of each test, and the associated reduced covariance matrix. Comparable values for the speed tests are shown in the bottom half of Table 6. The fairly simply calculations lead to estimated composite reliabilities of .910 and .959 for the verbal and speed sum scores, respectively. Of considerable interest, the composite reliabilities are reversed in magnitude for the verbal and speed sum scores—relative to values of coefficients *α* and *ω*_*T*_—due to the substantially higher reliabilities of the three speed tests in comparison to those for the three verbal tests.

**Table 6:** Reliabilities of verbal and speed composite scores based on scaled scores (x06–x13)

Variable names	Raw covariance matrix				Reduced covariance matrix			Composite
Verbal tests	x06	x07	x09	*r*_xx_	x06	x07	x09	Reliability
x06 Paragraph Compr	1.355	1.101	0.899	.700	0.949	1.101	0.899	
x07 Sentence Compl	1.101	1.665	1.018	.790	1.101	1.316	1.108	
x09 Word meaning	0.899	1.018	1.200	.860	0.899	1.018	1.032	
	Sum = 10.256				Sum = 9.332			.910
	Raw covariance matrix				Reduced covariance matrix			
Speed tests	x10	x12	x13	*r*_xx_	x10	x12	x13	
x10 Addition	1.187	0.537	0.375	.955	1.134	0.537	0.375	
x12 Counting dots	0.537	1.025	0.459	.930	0.537	0.954	0.459	
x13 S-C Caps	0.375	0.459	1.018	.885	0.375	0.459	0.901	
	Sum = 5.972				Sum = 5.729			.959

For variable names, Compr = comprehension; Compl = completion, S-C Caps = straight-curved capitals. *r*_xx_ = reliability, calculated as the average of reliability estimates for the Pasteur and Grant-White schools (Holzinger & Swineford, 1939). Reduced covariance matrix is identical to raw covariance matrix, except that error variance has been subtracted from each variance on the diagonal. Composite reliability = (sum of elements of reduced covariance matrix) / (sum of elements of raw covariance matrix).

## Using scores in subsequent analyses

### Sum scores versus factor scores: does it matter?

Another major argument by McNeish and Wolf (2020) was that use of sum scores versus presumably more precise factor scores can have unacknowledged differential impact when these scores are used in later analyses. In framing this argument, they distinguished multistage and simultaneous analytic approaches. In a multistage approach, the first step involves (a) performing a factor analysis and estimating factor scores or (b) calculating sum scores for individuals. In a second stage, the estimated factor scores or sum scores are used as predictors or as criteria in analyses with one or more external variables or covariates.

The other, simultaneous analytic approach is often conducted with structural equation modeling methods. Here, multiple items or indicators for each latent variable are specified, and relations among latent variables can be estimated within the context of a single-step analysis. Manifest (i.e., non-latent) covariates can also easily be incorporated in such models. In a simultaneous method, factor scores need not be separately estimated. That is, the means, variances, and covariances of latent variables and their relations with other variables can all be estimated within the simultaneously estimated structural model.

McNeish and Wolf (2020) noted that researchers should be wary of using sum scores, which might be relatively imprecise, rather than more precise factor scores, as alternate methods of scoring might lead to different conclusions in analyses of data. This is a serious concern and deserves considerable attention, which we illustrate with two empirical demonstrations.

### Demonstration #2: personality data and comparison of estimated factor and sum scores

Here, we compare two multistage analytic approaches, contrasting the correlations of estimated factor scores and sum scores with a set of criteria. We used an empirical data set, the spi data set (adapted from Condon, 2017), accessible through the psychTools package (Revelle, 2021b) in R (R Core Team, 2021). The data set contains responses by a sample of 4000 participants to a 135-item pool of personality items, and 10 criterion variables are also available. All personality items were answered on a 6-point scale, from 1 = “strongly disagree” to 6 = “strongly agree.” The 135 items consist of 27 narrow five-item scales, which can also be configured into five broader 14-item scales for the Big 5 dimensions of Extraversion, Agreeableness, Conscientiousness, Neuroticism, and Openness. The 10 criterion variables and their scoring/codes are:

- health, on a scale from 1 = “poor” to 5 = “excellent”;
- sex, coded 1 = male, 2 = female;
- exercise, scale from 1 = “very rarely” to 6 = “more than 5 times per week”;
- age, in years;
- education, from 1 = “less than 12 years” to 8 = “graduate or professional degree”;
- emergency room visits, from 1 = “none” to 4 = “3 or more times”;
- smoke, from 1 = “never” to 9 = “over 20 times per day”;
- parent 1 education, same scale as education (e);
- parent 2 education, same scale as education (e); and
- wellness, self-rated as 1 = “poor,” 2 = “good.”

Using the 14 items for the Neuroticism scale, we randomly selected 200 participants, extracted one factor from the 14 items, and used the factor scoring coefficients to estimate factor scores for the remaining 3800 participants. We also computed the simple unit-weighted sum of 14 Neuroticism items for the remaining 3800 participants. We then computed correlations of the estimated factor scores and the unit-weighted composite scores with the 10 criteria, and repeated this process 100 times. The resulting correlations are cross-validated validity correlations.

The cross-validated validity correlations for the 10 criteria are shown in Table 7. The mean correlation and the range of correlations (i.e., minimum and maximum) across the 100 samples are shown for the estimated factor scores in the first three data columns, and the corresponding values for the unit-weighted composites are shown in the last three data columns. Inspection of Table 7 reveals that, for each criterion, the mean correlation for estimated factor scores is virtually indistinguishable from the mean correlation for unit-weighted composites, and the same is true for the minimum and maximum correlation values. The largest difference was the correlation with sex, with a mean *r* = .25 for estimated factor scores and *r* = .24 for unit-weighted composites, a difference favoring estimated factor scores, but a difference of relatively small magnitude. The differences in mean and range of cross-validated correlations across the remaining nine criteria were extremely small. These results support the conclusion that little is to be gained from the use of factor score estimates rather than use of simpler unit-weighted sum scores when cross-validating results across samples.

**Table 7:** Cross-validated correlations of criteria with factor score estimates and unit-weighted composites for the neuroticism scale, across 100 random samples of 3800 participants

	Factor score estimates			Unit-weighted composites		
		Range			Range	
Criterion	Mean	Min	Max	Mean	Min	Max
Health	.34	.32	.35	.34	.33	.35
Sex	.25	.24	.27	.24	.23	.25
Exercise	.18	.17	.19	.18	.17	.19
Age	.18	.16	.19	.17	.16	.18
Education	.17	.16	.18	.17	.16	.19
Emergency room visits	.12	.11	.13	.12	.11	.13
Smoke	.05	.04	.06	.05	.05	.06
Parent 1 education	.05	.04	.06	.05	.04	.06
Parent 2 education	.04	.03	.06	.04	.03	.06
Wellness	.01	.00	.02	.02	.01	.03

### Demonstration #3: ability data and alternate methods of scoring

For our third and final demonstration, we return to the six manifest variables from Holzinger and Swineford (1939), comparing multistage and simultaneous analytic results using the same basic approach as McNeish and Wolf (2020). Given our concerns about precision of rounded scores, which McNeish and Wolf used, we conducted all analyses twice, once with rounded scores (r06–r13) and once with the more precise scaled scores (x06–x13). Results of the two sets of analyses exhibited minor differences but generally led to similar conclusions, so we reported here only results based on unrounded scaled scores. Relevant results using rounded scores are available in online Supplementary Material. All analyses were performed using the lavaan (version 0.6-9; Rosseel, 2021), psych (Revelle, 2021a), and psychTools (Revelle, 2021b) packages in R, and, where possible, replicated using Mplus 8.7 (Muthén & Muthén, 1998-2019).

The analyses reported by McNeish and Wolf (2020) involved using four methods for predicting performance on the six ability tests from School Membership, to contrast the use of sum scores versus more sophisticated factor scores in analyses. To save space, we refer readers to McNeish and Wolf (2020) for most details regarding the four methods, as we followed their lead in model fitting. In each of the four methods, School Membership (coded 0 = Pasteur, 1 = Grant-White) was the sole predictor variable. Briefly, the four methods were

- Method 1: Use an equally weighted sum of all six tests as the sole outcome variable.
- Method 2: Use equally weighted sums of the three Verbal tests and the three Speed tests as two separate outcome variables.
- Method 3: Perform a multistage factor score regression. Stage 1 involved fitting a two-factor congeneric factor model to the six variables and estimating factor scores. Stage 2 used School Membership as predictor of the estimated scores on the Verbal and Speed factors.
- Method 4: Perform a simultaneously estimated latent variable regression model, with a two-factor congeneric factor model fit to the six tests, and School Membership used as predictor of the two latent outcome variables, Verbal and Speed.

McNeish and Wolf (2020) conducted these analyses to contrast results and conclusions based on sum scores (Methods 1 and 2) versus more precise and rigorous scoring methods (Methods 3 and 4). If method of scoring moderates findings and affects conclusions drawn from analyses, use of simple sum scores should be scrutinized much more than is typically done.

Prior to presenting results, we note three issues. First, without explanation, McNeish and Wolf (2020) switched from ML estimation (used in their confirmatory factor analyses) to MLR estimation in these prediction models. MLR estimation is used to enable more robust estimation of certain parameters, notably standard errors (*SE*s) of parameter estimates, in the presence of non-normality of manifest variables. But skewness and kurtosis values listed for all ability variables in Table 2 were relatively small in size, suggesting that manifest variables were approximately normally distributed, implying that ML estimation would be an appropriate method of estimation. Hence, we decided to compare results under both MLR and ML estimation, to determine whether method of estimation might moderate findings.

A second issue is the evaluation of statistical significance. McNeish and Wolf (2020) relied solely on *p*-values. That is, if the *p*-level for a test statistic was .05 or less, a statistically significant finding was announced, whereas a finding with a *p*-level greater than .05 was rendered as indicating “no difference.” We prefer a more nuanced approach, more in line with Rosnow and Rosenthal (1989) who observed wryly that “… surely, God loves the .06 nearly as much as the .05.” Thus, we emphasize parameter estimates, *SE*s, and 95% CIs, rather than relying solely on whether a result falls on one side or the other of the .05 level of significance. To aid in evaluation of magnitude of effects, we report two forms of standardized estimates. If only dependent variables are standardized, standardized estimates are in the metric of Cohen’s *d* values, which convey group differences in mean performance in *SD* units. Thus, a *d* = 0.50 indicates that one group scores, on average, one-half a *SD* higher than the other group. If both outcomes and predictors are standardized, coefficients are in the metric of standardized regression coefficients, or *β* weights. Because School Membership is the only predictor in these analyses, the *β* weight is equal to the correlation (*r*) of School Membership with the outcome variable, and correlation is a recommended index of effect size (Funder & Ozer, 2019).

Third, Methods 1, 2, and 4 are routinely used in any structural modeling package, but Method 3 is implemented in easily usable form in only a few packages. We used the sam (or “Structural-After-Measurement”) fitting function in lavaan (Rosseel, 2021; Rosseel & Loh, 2021), which implements factor score regression. Skrondal and Laake (2001) gave an improved foundation for factor score regression, and Croon (2002) showed how to correct for bias in parameter estimates. More recently, Rosseel and associates (Devlieger et al., 2016: Devlieger & Rosseel, 2017; Devlieger et al., 2019; Rosseel & Loh, 2021) extended the Croon approach in multiple ways, by deriving *SE*s for parameter estimates, developing model fit indices and model comparison tests, and explicating various options in estimation. We used the “local” option with the default ML mapping matrix in the sam fitting function (see Rosseel & Loh, 2021), which is equivalent to the Skrondal and Laake approach with Croon’s correction and the recently derived *SE*s of parameter estimates. The sam approach has proven less susceptible to certain problems, such as lack of convergence and bias due to model misspecification, relative to simultaneous model estimation (i.e., Method 4), particularly when sample size is small, so is a worthy alternative to fully simultaneous model fitting. We also note that Method 3 shares with Method 2 the two-step analytic approach albeit with differential weighting of indicators and shares with Method 4 the use of sample-based differential weights. Thus, one might expect Method 3 to provide results that fall between those for Methods 2 and 4.

Results from our analyses are shown in Table 8, with results using MLR estimation in the top half of the table and results using ML in the bottom half. McNeish and Wolf (2020) argued that their results demonstrated that sum scores, under Methods 1 and 2, led to rather different results than more rigorous latent variable methods, Methods 3 and 4. Method 1 implied that Grant-White students scored higher in general, and Method 2 supported the contention that Grant-White students scored significantly higher on the Verbal factor and significantly lower on the Speed factor relative to Pasteur students. Methods 3 and 4 supported superior performance of Grant-White students on the Verbal factor, but McNeish and Wolf (2020) argued that results for Methods 3 and 4 supported a finding of “no difference” between schools on the Speed factor.

**Table 8:** Results of predicting ability outcomes from school membership using scaled scores (x06–x13)

				Standardized estimates			
Estimator and method	Outcome variable	Raw score estimates		Outcome variables		Outcomes and predictor	
		*B (SE)*	95% CI	*d* (*SE*)	95% CI	*β* (*SE*)	95% CI
MLR estimation							
Method 1	General	**1.08** (0.51)	[ 0.08, 2.08]	**0.24** (0.12)	[ 0.02, 0.47]	**0.12** (0.06)	[ 0.01, 0.23]
Method 2	Verbal	**1.76** (0.35)	[ 1.06, 2.45]	**0.55** (0.10)	[ 0.35, 0.76]	**0.28** (0.05)	[ 0.17, 0.38]
	Speed	**–0.68** (0.28)	[–1.23, –0.13]	**–0.28** (0.11)	[–0.50, –0.06]	**–0.14** (0.06)	[–0.25, –0.03]
Method 3	Verbal	**0.58** (0.12)	[ 0.35, 0.81]	**0.59** (0.11)	[ 0.37, 0.80]	**0.29** (0.06)	[ 0.18, 0.40]
	Speed	–0.16 (0.09)	[–0.34, 0.01]	–0.24 (0.13)	[–0.50, 0.02]	–0.12 (0.07)	[–0.25, 0.01]
Method 4	Verbal	**0.57** (0.12)	[ 0.34, 0.80]	**0.59** (0.11)	[ 0.37, 0.80]	**0.29** (0.06)	[ 0.19, 0.40]
	Speed	–0.22 (0.12)	[–0.46, 0.01]	**–0.31** (0.15)	[–0.60, –0.02]	**–0.16** (0.07)	[–0.30, –0.01]
ML estimation							
Method 1	General	**1.08** (0.51)	[ 0.09, 2.08]	**0.24** (0.11)	[ 0.02, 0.47]	**0.12** (0.06)	[ 0.01, 0.23]
Method 2	Verbal	**1.76** (0.36)	[ 1.06, 2.45]	**0.55** (0.11)	[ 0.35, 0.76]	**0.28** (0.05)	[ 0.17, 0.38]
	Speed	**–0.68** (0.28)	[–1.22, –0.13]	**–0.28** (0.11)	[–0.50, –0.06]	**–0.14** (0.06)	[–0.25, –0.03]
Method 3	Verbal	**0.58** (0.12)	[ 0.35, 0.81]	**0.59** (0.11)	[ 0.37, 0.80]	**0.29** (0.06)	[ 0.18, 0.40]
	Speed	–0.16 (0.09)	[–0.34, 0.02]	–0.24 (0.13)	[–0.50, 0.02]	–0.12 (0.07)	[–0.25, 0.01]
Method 4	Verbal	**0.54** (0.12)	[ 0.34, 0.80]	**0.59** (0.11)	[ 0.37, 0.80]	**0.29** (0.06)	[ 0.19, 0.40]
	Speed	**–0.22** (0.10)	[–0.42, –0.03]	**–0.31** (0.13)	[–0.57, –0.05]	**–0.16** (0.07)	[–0.29, –0.02]

Tabled values are parameter estimates with SEs and 95% CIs. *B* = raw score regression coefficient; *d* = estimate in Cohen’s d metric; *β* = standardized regression weight. Boldfaced coefficients had 95% CIs that did not include zero, so were significant at *p* < .05.

Contrary to McNeish and Wolf (2020), we argue that results under Method 1 offer no useful information at all in connection with their claims. As noted above, all one-factor models fit the data very poorly. Therefore, results under Method 1 are impossible to justify psychometrically, and all results under this method defy rational interpretation.

The remaining three methods—Methods 2, 3, and 4—each contained Verbal and Speed outcome variables, so were, at least on the surface, more similar in form. Each method identified a statistically significant advantage in favor of Grant-White students on the Verbal factor. Across Methods 2 - 4 and across estimators (MLR and ML), the mean difference on the Verbal dimension ranged between Cohen’s *d* values of about +0.55 and +0.60, so Grant-White students scored, on average, a little over one-half *SD* unit higher relative to Pasteur students.

Each of the three methods also identified a mean difference of more modest magnitude favoring Pasteur students on the Speed factor, and the standardized estimates across Methods 2, 3, and 4 were quite similar. Across methods, *d* values ranged from –0.24 to –0.31, or about a 0.3 *SD* advantage for Pasteur students, and the associated 95% CIs sometimes included zero, and sometimes did not, even though the magnitudes of the *d* values differed little across methods.

Hence, contrasts among methods do not support a simple conclusion that the raw score method (Method 2) led to notably different results than latent variable methods (Methods 3 and 4). Indeed, the multistage sum score method, Method 2, produced results that were very similar to the simultaneously estimated latent variable approach, Method 4. Point estimates under Method 4 were slightly larger than those under Method 2, which might be expected, given that Method 4 was a latent variable method testing school effects at an error-free level, whereas Method 2 tested school effects at a manifest variable level. Regardless, the point and interval estimates for standardized *d* and *β* estimates for both the Verbal and Speed dimensions were very similar in magnitude across Methods 2 and 4, especially under ML estimation.

The method that appeared to provide the most different results was Method 3. The factor score regression approach produced slightly smaller raw and standardized point estimates for the Speed factor than did its simultaneously estimated, latent variable cousin, Method 4. Given the smaller point estimates, the 95% CIs for Speed factor parameter estimates included zero, so the point estimates did not depart from zero at *p* = .05. Thus, the most striking difference among the three methods was that between Method 3 versus Methods 2 and 4.

We encourage researchers to analyze their data using different approaches to determine whether alternate methods yield results that lead to different conclusions. If discrepant results are obtained using alternate methods, the researcher must provide a compelling justification for the choice of the method to emphasize and should report all results in some fashion.

### Discussion

Basic aspects of measurement must become the subject of greater attention if the science of psychology is to progress at maximal speed. The use of equally weighted sums of scores on a set of items is potentially fraught with problems that remain implicit if unexamined. The sum of a set of items seems like such a simple operation, but dangers lurk below the surface if the sum score is not completely understood psychometrically. If a scale is composed of a heterogeneous set of items, relations of scale scores with external variables may depend on different subsets of items across studies. This may be an important problem contributing to the replication crisis confronting psychology and other social sciences.

As many recent commentators have noted, important decisions must be made when using a multiple-item scale. Is a single sum of the set of items justified? Should item scores be treated as discrete or continuous? Were scores on negatively worded items properly reverse-scored? Does the set of items represent more than one latent factor? If the scale is multidimensional, how should this information be used when deciding how to sum sets of items? If items have rather different variances, should item scores be standardized in some fashion before being summed? Are item scores approximately normally distributed? If not, should some kind of transformation be made prior to summing the items? Greater care in making decisions on these questions is warranted, researchers should be fully transparent in reporting exactly how they dealt with each issue (Flake & Fried, 2020), and this should lead to more informative and replicable research outcomes. If the principal aim McNeish and Wolf (2020) was to alert researchers to these important issues, we would applaud this message.

But McNeish and Wolf (2020) went much further than this, asserting that summing the scores of multiple items implies that a researcher must assume that a highly constrained parallel test model with equal loadings on a single factor provides close fit to the covariances among the items. Directly, McNeish and Wolf (2020) stated that the use of sum scores “… obliges researchers to engage with model constraints they are imposing (perhaps unknowingly) and test the assumptions associated with such constraints.” This assertion is simply and blatantly false. The key criterion to be met to justify use of a sum score—from a homogeneity perspective—is that a one-factor model provides an adequate description of the relations among the items. If a one-factor essentially parallel test model or a one-factor essentially tau equivalent test model fits the item data adequately, then a number of formulas can be used to estimate the reliability of the equally weighted sum of items, including the formula for coefficient *α* (Eq. 5), and many computer programs readily report such estimates. However, if a highly constrained model does not achieve adequate fit, and a one-factor congeneric model does provide adequate fit to the data, an equally weighted sum of item scores is still justified, but Eq. 5 is no longer a recommended estimator of reliability. Instead, one should use a more appropriate equation, such as Eq. 7 for coefficient *ω*_*T*_, to estimate reliability of the sum score. If a one-factor model does not fit the data adequately and two or more factors are required, more complex approaches must be taken to estimating reliability, and much current work has been working out these details (e.g., Revelle & Condon, 2019; Revelle & Zinbarg, 2009). But, to repeat, using a sum score across a set of items does not mean that a researcher assumes implicitly, usually without knowing, that a very highly constrained factor model must fit the data. Estimates from an essentially unconstrained, congeneric one-factor model, if it fits the data closely, can be employed to separate true score variance from error variance in the set of items with Eq. 7, allowing estimation of reliability for the equally weighted sum of items (cf. Eq. 4).

If one were to use a differentially weighted sum of item scores, it is possible to estimate reliability of the resulting summary score. Moreover, reliability of a differentially weighted sum may be somewhat higher than the reliability of an equally weighted sum of item scores, especially if the differential weights vary considerably. However, increases in reliability may not be accompanied by appreciable increases in validity and, especially, in cross-validated validity, so this should be the topic for active investigation and determination.

In support of the use of sum scores, we reiterate a point made earlier, *if sum scores are composed in exactly the same fashion across studies, the results of the studies will be more easily compared than if different weighting were used across studies*. As noted earlier, this advantage in favor of sum scores holds only if no changes to item content (e.g., wording) and administration are made across studies. If one opts for a method of differential weighting of item scores in a given study, results of that study will be comparable to other studies only if an identical set of differential weights were used to sum items in the other studies.

To promote their arguments in favor of factor scoring methods over use of sum scores, McNeish and Wolf (2020) presented analyses of HS data using different analytic methods. In doing so, McNeish and Wolf engaged in what we characterize as questionable measurement practices and propounded questionable interpretations. One questionable measurement practice was rounding scaled scores from the HS data set to integer values. Whether conclusions would be altered by using rounded, less precise scores in analyses is a matter for research, but rounded scores clearly have less precision than scaled scores, and this practice should not be condoned. A second questionable practice is the sole reliance on *p*-values when evaluating mean differences across groups, rather than using estimates of magnitude of effects and their CIs.

Perhaps the most problematic interpretation by McNeish and Wolf (2020) was that their results provided clear support for latent variable modeling over simpler and more mundane sum scoring of indicators. Under greater scrutiny, this claim cannot be supported. The sum score method, Method 2, yielded results that were extremely similar to those for the standard SEM method using simultaneous fitting, Method 4. The method that provided the most discrepant results was Method 3, using factor score regression. Still, on balance, we argue that all three methods—Methods 2, 3, and 4—led to results that were very similar, and no claim that latent variable methods are superior to sum score methods is justifiable on the basis of these results.

The use of sum scores may not always produce results that are virtually indistinguishable from those based on latent variable modeling. If sum scores are based on a rather small number of scores to be summed and if those scores are single items, the resulting sum score might have rather low levels of reliability and this could lead to notable differences across analytic methods. In our analyses, the Verbal and Speed sum scores had very high levels of composite reliability, .91 and .96, respectively. The very high reliability of the sum scores used in our analyses is probably why sum scores and fully latent variable modeling under Method 4 led to such similar results. This, clearly, is an issue for further research.

One should note that Method 3 was touted by McNeish and Wolf (2020) as a latent variable method, but its multistage nature leads to factor scores under this method that are, in essence, quite similar to sum scores (i.e., amalgams of true score and error variance) but obtained under differential (rather than equal) weighting. The Croon correction for bias in estimated factor scores was a notable advance, and the recent work by Rosseel and colleagues in providing *SE*s of parameter estimates and other enhancements and implementing these methods in the open source lavaan package in R provide easily usable methods in furthering research on the relative advantages and disadvantages of sum scores and estimated factor scores.

In the current study, the simple equally weighted Verbal and Speed sum scores in Method 2 yielded results that appeared to be slightly more robust across methods of estimation—MLR vs. ML—than the simultaneous estimation of latent variable scores under Method 4. As for sensitivity, although Method 4 led to somewhat larger estimates of standardized effect size than did Method 2, as expected, differences were not large. In summary, contrary to conclusions by McNeish and Wolf (2020), sum scores appear to be as strong a basis for analyses as are complex latent variable procedures in analyses of the HS data.

Note that this conclusion is fully in line with our results of the analyses of spi data reported in Table 7. In these analyses, we contrasted the magnitude of correlations of factor score estimates and simple sum scores with 10 criteria. The upshot of these analyses is that the correlations of the factor score estimates with the criteria were virtually indistinguishable from the comparable correlations of the sum scores with those criteria. That is, factor score estimates and sum scores had essentially equivalent cross-validated validities, with no notable superiority of either method. This underscores our contention that sum scores may often be as strong a basis for psychological research as are more complicated latent variable methods.^7^

Factor analytic methods should be used to represent the internal structure of items comprising a scale, and the resulting factor structure should be the basis for deciding how items are composited into scores to be used in subsequent analyses. Factor-based methods of estimating reliability are easy to apply (e.g., the omega function in the psych package) and computer scripts for estimating more recent proposed methods by Rae (2007) and Nicewander (2020) are provided in Supplemental Material. But, when forming scores, sum scores are decidedly simpler to implement than are factor score estimation methods, which may be a notable advantage in certain situations, such as when cross-validating results across independent samples. On the other hand, sum scores are likely to contain larger amounts of measurement error (or unreliability) than will estimated factor scores and certainly more than the error-free (so-called) true factor scores in confirmatory factor models. So, sum scores, estimated factor scores, and true factor scores (the latter residing in the computations in structural equation models) each have strengths and each have weaknesses, and none outshines the others in all respects. As measurement precision and, hence, reliability of measures increase, results using the different methods will tend to converge on the same answers. We urge researchers to measure their constructs as well as possible and to conduct analyses using multiple methods to help ensure that conclusions drawn are robust across analytic methods.

Long live sophisticated latent variable methods! But, long live sum scores (properly vetted)!

## Supplementary Information

ESM 1 (DOCX 28 kb)

ESM 2 (DOCX 164 kb)

## Footnotes

1 By sum scores, we mean either the sum of responses to items in a scale or to the mean item response across the scale items. With complete data, sums and means of item responses clearly are equivalent estimates, with sum scores confounding the number of items with the mean item responses. Mean scores have the advantage of being in the measurement units of the items. With missing data, sum scores conflate missingness with 0 (unless complex corrections are used), whereas mean scores implicitly impute a person’s mean item response for missing data. Sum scores are more common in some subdisciplines of psychology, mean scores in others.

2 Technically, within a common factor model, the residuals, the *ε*_*j*_s, are scores on unique factors, so use of a term such as *errors* can be misleading. The variance of a unique factor is an additive combination of specific variance (i.e., reliable variance not shared with the common factor or latent variable) and random measurement error variance. Hence, to label the variance of a residual as error variance tends to gloss over the distinctions afforded unique factors. To maintain comparability in wording with McNeish and Wolf (2020) we will use the term *error variance*, but note that this error variance includes both reliable variance specific to the indicator plus random error.

3 Many presentations of coefficient omega use the simple ω symbol for this statistic. We have chosen to use the symbol *ω*_*T*_to signify that we are presenting a coefficient that reflects the proportion of total variance that is due to all common factors. If only a single factor underlies a set of items, *ω*_*T*_ represents the proportion of scale variance due to that one factor. But, if multiple factors contribute to a set of items (e.g., leading to a bifactor structure), *ω*_*T*_ represents the proportion of scale variance across all factors.

4 Both parallel test and tau equivalent test models have equal loadings on a single factor, so both can reproduce equal covariances among items and both lead to estimated factor scores that correlate perfectly with sum scores. But whether a parallel test or tau equivalent test model provided good fit to an interitem covariance matrix was never discussed by Cronbach (1951). Derivations by Cronbach were based on the myriad ways to form split-halves of a set of items, where the various split-halves were sum scores of randomly allocated, non-overlapping sets of items, and item covariances were assumed to be positive, but often unequal. Cronbach stressed the importance for *α* of one major factor underlying a set of items, but never dealt in any detail with the issue of equality of loadings on the factor. Indeed, because Cronbach presented results for which coefficient *α* varied across random split-halves, the loadings on the single major factor were implicitly assumed to be unequal. Discussion of parallel tests and their characteristics predated Cronbach (1951), but tau equivalent tests are a more recent innovation. An APA PsycInfo search (on 19 April 2021) for the terms “tau equivalent” or “tau equivalence” found that the first use of “tau equivalent” in a refereed publication appears to have been by Jöreskog (1971), who attributed the term to Lord and Novick (1968). Furthermore, usable statistical software to specify, estimate, and test the fit of parallel and tau equivalent models only became available about two decades after Cronbach (1951), with the contribution by Jöreskog (1971). Thus, parallel or tau equivalent test models are not the basis for the initial derivation of coefficient *α*, but were recognized more than a decade later as the implicit assumption required so that coefficient *α* yields a justified model-based index of reliability.

5 All properties of sum scores, including reliability, are also true of mean item scores (or the average score across a set of items). The mean item score is a simple linear transformation of a sum score, obtained by dividing the sum score by the number of items, and has the interpretive advantage of falling on a scale with the same units as that on which each item was rated.

6 As discussed in Supplemental Material, we used the letter ‘t’ to designate scores on the six tests in their raw score form, the letter “x” to designate linearly rescaled or transformed scores on the tests, and the letter “r” to indicate rounded versions of the rescaled (or transformed) scores on the tests. We also used numbers to indicate the position of a given test in the Holzinger and Swineford (1939) battery. Thus, t06, x06, and r06 refers to paragraph comprehension test scores in raw, scaled, and rounded form, respectively; t07, x07, and r07 to raw, scaled, and rounded scores, respectively, on sentence comprehension, etc. In the current manuscript, we present analyses of the scaled scores, to maintain score accuracy consistent with analyses reported by Holzinger and Swineford. In Supplemental Material, we compared analytic results using scaled scores with results based on rounded scores (used by McNeish & Wolf, 2020) to identify any notable differences that arose from rounding of scores.

7 Comparison of the relative strengths and weaknesses of estimated factor scores and sum scores when investigating mean differences across groups was considered beyond the scope of the present article. This topic is discussed in some detail in Supplemental Material, in connection with the R script titled “prog_02.sex diffs on factor scores vs. sum score.R”.

## Declarations

### Prior versions

NA

### Preregistration

No preregistration was required for this paper as it did not involve the conducting of new empirical studies, but relied on archival data.

### Data, materials, & online resources

All raw data analyzed in the manuscript are available through the psychTools package in the R computing environment. R script files require access to the psych, lavaan, and psychTools packages in R. Supplemental information and the R script files that show how to access data and then run all analyses are provided on an Open Science Framework page, located at https://osf.io/tev6g/

### Reporting

This study involved publicly available archival data; no new data were collected.
