# Supplementary material for: Thinking thrice about sum scores, and then some more about measurement and analysis
Source: Behav Res Methods. 2022 Apr 25;55(2):788–806. doi: 10.3758/s13428-022-01849-w (PMC10027776; doi:10.3758/s13428-022-01849-w)
Supplement: Supplementary file 1 — (DOCX 28 kb) [file 13428_2022_1849_MOESM1_ESM.docx]

Supplemental Material to be available online (not for publication)

to accompany

**Thinking thrice about sum scores, and**

**then some more about measurement and analysis**

This is a “read me” file to serve as introduction to online material to supplement the abovenamed manuscript. The following supplemental materials are available:

**I. Document titled “Supplemental Material. Tables and such”**

This document contains useful information in a number of sections, as follows:

**Detailed Notes on Holzinger-Swineford Data: Dos and Don’ts**

The first section of the Supplemental Material contains many details about the nature of the Holzinger-Swineford data. In particular, the descriptive statistics for the raw scores, scaled scores, and rounded scaled scores for the six tests used by McNeish and Wolf are provided. We also demonstrate that the rounded scores have poorer psychometric properties, implying that rounding of scores (as McNeish and Wolf did) led to degraded information in the scores.

**Comparison: Estimated Factor Scores vs. Sum Scores for Investigating**

**Mean Differences across Groups**

In this section, comparisons and contrasts are presented with regard to the use of estimated factor scores vs. sum scores when investigating mean differences across groups. Each approach – estimated factor scores and sum scores – has advantages, each has disadvantages. These are discussed briefly and then an empirical example is presented to exemplify some of the issues.

**Supplemental Tables**

This section contains a set of tables that supplement material in the main manuscript. The first two tables contain indices of fit for factor analytic models fit to scaled scores and to rounded scores. The next two tables present results for one-factor and two-factor models and for bifactor models fit to the scale scores and then the rounded scores. Two tables report results of analyses of rounded scores – one table giving alpha and omega coefficients, and the other table composite reliability estimates. The remaining tables provide results of different ways of testing the effect of school on dimensions of Vrbal and Speed ability from the HS data set.

**SAS Program Scripts for Bifactor Models**

An SAS program is included that shows how to calculate coefficient $\omega_{H}$and $\omega_{T}$ from the bifactor model estimates shown for “rounded” scores in Supplemental Table S6 and for “unrounded” scaled scores in Supplemental Table S5. Of note, the index of total reliability from coefficients $\omega_{H}$and $\omega_{T}$ show that reliability from rounded scores is lower than reliability from the unrounded scaled scores, underscoring our contention that rounding of scores led to discarding of reliable information.

**SAS Program Scripts to Estimate Reliability in Various Ways**

This file also contains SAS programs that demonstrate how to use results from Nicewander (2019) to calculate (a) the correlation of estimated factor scores with true factor scores (the square of which is the reliability of estimated factor scores); (b) the correlation of sum scores with true factor scores (the square of which is the reliability of sum scores); and (c) the correlation of estimated factor scores with sum scores. The SAS programs also provide code for estimating composite reliability, following methods presented by Rae (2007). The SAS programs used only the scaled scores and based calculations only on the Verbal and Speed scores.

These calculations are performed for the one-factor congeneric factor for six manifest variables (see manuscript Table 4) and for the Verbal and Speed factors (see manuscript Tables 5 and 6).

**II. Standalone R script files**

These R script files are placed on the Open Science Framework folder for the manuscript, for easy download and running of the scripts. Some script files are for analyses reported in earlier versions of the manuscript, but are informative nonetheless.

prog_01. fac score vs. sum scores with N.R

This program implements the analyses reported as cross-validated correlations of Neuroticism scale estimated factor scores and sum scores with 10 criteria reported in manuscript Table 7

prog_02. sex diffs on factor scores vs. sum score.R

This program estimates sex differences on the Neuroticism scale, based on estimated factor scores versus sum scores

prog_03. factor models.R

This program accesses the Holzinger and Swineford (1939) data and performs all factor models for “rounded” scores (cf. fit indices in Supplemental Table S4) and “unrounded” scaled scores (cf. fit indices in manuscript Table S3)

prog_04. predictions from school for R_version3.6.R

This program accesses the Holzinger and Swineford data and then performs predictions of ability test performance using four methods, as described in by McNeish and Wolf (2020). In this program, Method 3 – factor score regression – was performed using the fsr function in lavaan, in line with procedures used by McNeish and Wolf. The fsr function in lavaan ran successfully in R versions up to 3.6, but is no longer functional in R versions 4.0 and following.

prog_05. predictions from school for R_version4.0.R

This program accesses the Holzinger and Swineford data and then performs predictions of ability test performance using four methods, as described in by McNeish and Wolf (2020). Here, Method 3 – factor score regression – was performed using the sam function in lavaan, which is operational in the most up-to-date R versions 4.0 and following.

prog_05a. predictions from school for R_version4.0.R

This program, numbered 5a, provides identical results for the prediction analyses as does the preceding program. However, the initial sections of the program were revised by William Revelle to be more efficient and less cumbersome, utilizing the psych package in R.

prog_06. easy program for alpha and omega.R

This program accesses the scaled scores, identified as x4 through x9, that are resident in the lavaan program in R, and then uses the alpha and omega procedures from the psych package to obtain measures of reliability. The bifactor results are based on exploratory factor procedures (e.g., rotation of first-order factors, Schmid-Leiman transformation), so may differ slightly from results presented in SAS program for omega.
