# Supplementary material for: Thinking thrice about sum scores, and then some more about measurement and analysis
Source: Behav Res Methods. 2022 Apr 25;55(2):788–806. doi: 10.3758/s13428-022-01849-w (PMC10027776; doi:10.3758/s13428-022-01849-w)
Supplement: Supplementary file 2 — (DOCX 164 kb) [file 13428_2022_1849_MOESM2_ESM.docx]

Supplemental Material to be Available Online (not for publication)

to Accompany

**Thinking Thrice about Sum Scores, and**

**Then Some More about Measurement and Analysis**

Detailed Notes on the Holzinger and Swineford (1939) Data: Dos and Don’ts . . . . . . Page 2

Comparison: Estimated Factor Scores vs. Sum Scores for Investigating

Mean Differences across Groups . . . . . . . . . . . . . . . . . . . . . . Page 9

Supplemental Tables . . . . . . . . . . . . . . . . . . . . . . . . . . Page 12

SAS Computer Program to Compute Coefficient *ω* for Bifactor Models . . . . . . Page 23

SAS Programs for Estimating Reliability of Sum Scores and Estimated Factor Scores:

Nicewander (2020) Approach, Composite Reliability, and Selected Output . . Page 30

**Detailed Notes on Holzinger-Swineford (1939) Data: Dos and Don’ts**

**The Holzinger-Swineford (1939) Data**

McNeish and Wolf (2020) used six variables in their empirical examples. These variables are selected from a larger battery of 26 tests from the Holzinger and Swineford (1939) monograph, a data set long used in psychometric treatises. Harman (1976) used most of the manifest variables in the Holzinger-Swineford (hereinafter, HS) data protocol for his “24 psychological variables” example in his text on factor analysis. Meredith (1964a, 1964b) used a subset of HS data in his work on factorial invariance across multiple groups using exploratory factor methods, and Jöreskog (1969, 1971) followed up Meredith, extending the ideas to the confirmatory factor context. These are only a few of the publications that have exploited the HS data, which has become one of the most used data sets in the psychometric literature.

Rosseel (2019) refers to these six variables as x4 through x9 (comprising six of nine variables he uses in examples for use of lavaan), but they are actually tests 6, 7, 9, 10, 12, and 13, respectively, of the 26 tests in the HS protocol. Basic descriptive statistics on raw scores on these six variables are provided in the first six data rows of Table S1. [The original raw-scored data are available in the psychTools package in R as *holzinger.raw*]. We identify the original raw-scored versions of these six variables with a “t” to signify the *test* in the original Holzinger and Swineford (1939) battery and a number to indicate the ordinal position of the test in the battery. Hence, t06 is Paragraph Comprehension, the sixth test in the HS data battery. As seen in Table S1, t06 had a *M* = 9.18, *SD* = 3.49, and a range from 0 to 19. Because t06 consisted of 20 questions to be answered after reading several short paragraphs, the range of obtained scores spanned almost the total number of points possible. Descriptive statistics for raw scores on the remaining tests—t07, t09, t10, t12, and t13—reveal very different means, *SD*s, and score ranges. Note also that the minimum and maximum scores on each test are integer values, as test scores represent the number of items correctly answered on each test.

The middle set of six data lines in Table S1 provide the descriptive statistics for what we call *scaled* scores, and we use an “x” in the name for these scores to be consistent with the “x” designation for variables in the lavaan package. (Scaled scores for the HS data are available in the psychTools package in R in a data set called *holzinger.swineford*, along with descriptions on how rescaling was performed.) A bit of psychometric sleuthing revealed that the six “x” variables are simple linear transformations of the six raw scored “t” versions. Specifically,

$$\text{x06 = }\frac{\text{t06}}{3} \text{x07 = }\frac{\text{t07}}{4} \text{x09 = }\frac{\text{t09}}{7} \text{x10 = }\frac{\text{t10}}{23} \text{x12 = }\frac{\text{t12}}{20} \text{x13 = }\frac{\text{t13}}{36}$$

No basis for these very different linear transformations across tests were provided in the lavaan manual, which stated that the scores were obtained from a website that no longer has an active link. It appears that a specific divisor was selected for each test so the resulting scaled (or transformed) scores would have a maximal range from 0 to 10 and have much more similar means and *SD*s than was the case for the raw scored versions. Because scaled versions are linear transformations of raw scored versions and because maximum likelihood (ML) estimation is a scale-free technique, many statistical models—in particular, models that do not invoke equality constraints on factor loadings across indicators—will have identical fit to the raw-scored and scaled versions of these test scores under ML estimation, so rescaling did not alter basic information on the tests. But, minimum and maximum values for each test are no longer integer values, because the ratio rescaling of scores on variables led to informative decimal values.

Without clear justification, McNeish and Wolf (2020) then rounded the scores of the scaled variables to integer values (or whole numbers). In the psychTools package help page for the *holzinger.swineford* data set, we note that ratio transformations of the raw scores of tests in the HS data set had been used to rescale original raw scores into scores with approximate 0-10 point ranges (with at least seven decimals of precision, if needed). In the bottom six data rows of Table S1, descriptive statistics for the rounded scores are shown, using the letter “r” to identify the *rounded* versions of the scaled scores. The rounding of scaled scores did not alter the means and SDs of variables appreciably, although the minimum and maximum values are now integer values, due to the rounding. Moreover, care must be taken when rounding values. Certain “round” functions in R have unusual characteristics. When we first tried to round scaled scores, we could not reproduce precisely results reported by McNeish and Wolf. We found that the “round” command we used would round values with decimal values of exactly .50 to the nearest even integer, so 5.50 would round up to 6, but 6.50 would round down to 6.

We solved this problem by adding a small value (.001) to scaled scores, and rounding these slightly altered scaled values led to exact replication of results reported by McNeish and Wolf (2020). We caution researchers to be wary of computer programs and their internal workings. Intricacies such as these may be yet another reason why results fail to replicate across studies in the social sciences. Indeed, problems with rounding are perhaps one of the most frequently asked questions of R.

The first three variables—t06, t07, and t09—were power tests of Verbal Comprehension, signified as the V factor in work by Thurstone and colleagues (e.g., Thurstone, 1938; Thurstone & Thurstone, 1941). The test names provide reasonable indicators of the nature of the tests: t06 — Paragraph comprehension, participants answered 20 questions based on information in six paragraphs; t07—Sentence completion, 30 sentences, on each of which the participants were asked to select the single word of four options that best completed the sentence; T09—Word meaning, 50 items, on each of which the participants were asked to select the one of five option words that was the closest synonym for an underlined word in a sentence. As power tests, time limits for these tests were sufficiently liberal so that each participant was expected to have time to answer each question. Individual differences on these tests were, therefore, indicative of the ability of a participant to understand verbal information, with higher scores associated with higher levels of understanding or more advanced comprehension of English words and prose.

In contrast, the remaining three variables—t10, t12, and t13—were highly speeded tests, indicators of a Perceptual Speed factor in work by Thurstone (1938). These tests had the following form: t10—Addition, containing a large number of simple addition problems (i.e., addition of two single-digit numbers) and students were asked to write the correct sum; t12—Counting dots, consisting of a large number of squares each containing from 4 to 7 dots, with students to write down the number of dots in each square; t13—Straight-curved capitals, with a large list of capital letters, with participants instructed to write an underscore under capital letters constructed only of straight lines (e.g., E, F, M) or a circle under letters that had one or more curved lines (e.g., D, O, S). These three tests have very easy items, so liberal time limits would have resulted in perfect scores for virtually all students. With short time limits imposed, individual differences are reflected in speed and accuracy of performing the simple tasks. Hence, both the time limits and content—and thus the constructs to be assessed—differed markedly across the two sets of tests, so a one-factor model would not be expected to fit this data set well.

McNeish and Wolf (2020) reported results from analyses of school differences on dimensions underlying the six ability tests. Holzinger and Swineford (1939) collected data from seventh and eighth graders in two schools: Pasteur, a school in Chicago; and Grant-White, a school in a Chicago suburb. In the HS data set, the first 156 participants (with School coded 0) were from Pasteur school, and the remaining 145 students (coded 1) were from Grant-White. Thus, positive relations of the School variable with ability outcomes would indicate superior performance by Grant-White students. This is of interest, given the differing characteristics of the schools and their students. Holzinger and Swineford obtained family information from about 60% (93 of 156) of students in the Pasteur school, and an unspecified number of students from Grant-White. For the Pasteur school, many parents were workers in nearby factories. Both parents were foreign born in about half of the families (48%), in less than a third of families (29%) were both parents US born, and many families used the language of their country of origin at home. In contrast, for the Grant-White school, in only 15% of families were both parents foreign born, and in 72% of families both parents were US born. Thus, Pasteur students had a much larger immigrant representation than Grant-White, so Holzinger and Swineford expected Grant-White students to exhibit higher levels of mean performance especially on verbal tests, even though the Grant-White students were, on average, 6 months younger than Pasteur students.

At one point, McNeish and Wolf (2020) argued that scores—such as estimated factor scores and sum scores—should correlate at least *r* = .99 to claim that the scores, at the level of individuals, provided essentially comparable information. In Table S2, we provide correlations among scaled scores below the diagonal, correlations of rounded scores (used by McNeish and Wolf) above the diagonal, and the correlation of the scaled score on each test with its rounded counterpart on the diagonal. Correlations among rounded scores are lower than those among scaled scores, so reliability estimates from rounded scores would tend to be lower than those based on scaled scores, given the loss of information due to rounding of scores. Also, not one of the tests had rounded scores that correlated at least *r* = .99 with the more accurate scaled scores for the test, so critical information clearly was lost in the unfortunate rounding of test scores to integer values, casting doubt on the wisdom of rounding the scaled scores.

References

Harman, H. H. (1976). *Modern factor analysis* (3^rd^ ed). University of Chicago Press.

Holzinger, K. J., & Swineford, F. (1939). A study in factor analysis: The stability of a bi-factor solution. *Supplementary Educational Monographs*, No. 48. University of Chicago Press

Jöreskog, K. G. (1969). A general approach to confirmatory maximum likelihood factor analysis. *Psychometrika*, *34*, 183-202.

Jöreskog, K. G. (1971). Simultaneous factor analysis in several populations. *Psychometrika*, *36*, 409-426.

Meredith, W. (1964a). Notes on factorial invariance. *Psychometrika*, *29*, 177-185.

Meredith, W. (1964b). Rotation to achieve factorial invariance. *Psychometrika*, *29*, 187-206.

Thurstone, L. L. (1938). Primary mental abilities. *Psychometric Monographs*, No. 1.

Thurstone, L. L., & Thurstone, T. G. (1941). Factorial studies of intelligence*. Psychometric Monographs*, No. 2.

Table S1

Descriptive Statistics for Tests from Holzinger and Swineford (1939)

| Item | Description | *M* | *SD* | Skew | Kurt | Min | Max |
| --- | --- | --- | --- | --- | --- | --- | --- |
| Original raw scoring | |  |  |  |  |  |  |
| t06 | Paragraph comprehension | 9.18 | 3.49 | 0.27 | 0.08 | 0 | 19 |
| t07 | Sentence completion | 17.36 | 5.16 | −0.35 | −0.55 | 4 | 28 |
| t09 | Word meaning | 15.30 | 7.67 | 0.86 | 0.82 | 1 | 43 |
| t10 | Addition | 96.28 | 25.06 | 0.25 | −0.31 | 30 | 171 |
| t12 | Counting dots | 110.54 | 20.25 | 0.53 | 1.17 | 61 | 200 |
| t13 | Straight-curved capitals | 193.47 | 36.33 | 0.20 | 0.29 | 100 | 333 |
| Scaled (or transformed) scores | |  |  |  |  |  |  |
| x06 | Paragraph comprehension | 3.06 | 1.16 | 0.27 | 0.08 | 0.00 | 6.33 |
| x07 | Sentence completion | 4.34 | 1.29 | −0.35 | −0.55 | 1.00 | 7.00 |
| x09 | Word meaning | 2.19 | 1.10 | 0.86 | 0.82 | 0.14 | 6.14 |
| x10 | Addition | 4.19 | 1.09 | 0.25 | −0.31 | 1.30 | 7.43 |
| x12 | Counting dots | 5.53 | 1.01 | 0.53 | 1.17 | 3.05 | 10.00 |
| x13 | Straight-curved capitals | 5.37 | 1.01 | 0.20 | 0.29 | 2.78 | 9.25 |
| Rounded scaled scores | |  |  |  |  |  |  |
| r06 | Paragraph comprehension | 3.09 | 1.17 | 0.24 | 0.24 | 0 | 6 |
| r07 | Sentence completion | 4.47 | 1.33 | −0.28 | −0.62 | 1 | 7 |
| r09 | Word meaning | 2.20 | 1.13 | 0.83 | 0.76 | 0 | 6 |
| r10 | Addition | 4.20 | 1.15 | 0.13 | −0.44 | 1 | 7 |
| r12 | Counting dots | 5.56 | 1.03 | 0.59 | 0.97 | 3 | 10 |
| r13 | Straight-curved capitals | 5.37 | 1.08 | 0.16 | 0.23 | 3 | 9 |

*Note*: Elements in the Item column identify manifest variables, where “t” stands for “test” as originally scored, “x” for the scaled (or transformed) versions as provided by the lavaan program, and “r” for the rounded versions of scaled scores. The numbers (06, 07, etc.) refer to the ordinal position of tests in the original Holzinger & Swineford (1939) protocol and monograph.

Table S2

Correlations among the Six Tests from Holzinger and Swineford (1939)

|  | ParaComp | SentComp | WordMean | Addition | CountDot | SCCaps |
| --- | --- | --- | --- | --- | --- | --- |
| ParaComp | (.97) | .70 | .66 | .15 | .07 | .20 |
| SentComp | .73 | (.98) | .67 | .10 | .17 | .21 |
| WordMean | .70 | .72 | (.97) | .12 | .14 | .18 |
| Addition | .17 | .10 | .12 | (.97) | .42 | .30 |
| CountDot | .11 | .14 | .15 | .49 | (.96) | .40 |
| SCCaps | .21 | .23 | .21 | .34 | .45 | (.96) |

*Note*: Correlations above the diagonal are based on rounded scaled scores used by McNeish and Wolf (2020); correlations below the diagonal are from scaled scores as available in lavaan; correlations on the diagonal (in parentheses) are correlations between the scaled and rounded scores. ParaComp = Paragraph Comprehension, SentComp = Sentence Completion, WordMean = Word Meaning, CountDot = Counting Dots, SCCaps = Straight-Curved Capitals.

**Comparison: Estimated Factor Scores vs. Sum Scores for Investigating**

**Mean Differences across Groups**

Mean differences across groups can be investigated in several ways, as discussed in the manuscript. For example, if one were interested in the mean difference between males and females on a particular personality factor, one could test the mean difference between groups using estimated factor scores or sum scores as dependent variable. The point of this note is to discuss some strengths and weaknesses of each approach.

**Estimated factor scores**. A researcher could perform a factor analysis of items from the scale and use these results to estimate scores on the factor underlying the set of items. The researcher could choose either (a) to perform one factor analysis including persons from both groups in a single analysis, or (b) to perform separate factor analyses in each group. After choosing one of the two options, factor scores could be estimated based on the factor analytic results.

Taking option (a) to perform a single factor analysis using data from all participants (i.e., across both groups) has some strengths and some weaknesses. One strength of this approach is that a single set of compositing weights would be used for all participants, so the weighted scores are calculated using the same weights across groups. This approach does, however, have the disadvantage that substantial group mean differences on the manifest variables can lead to bias in the factor analytic results. That is, to the extent that groups differ in mean level on the factor, this mean difference will be confounded in the correlations among the manifest variables, so that the factor analytic results would be unlikely to replicate in a future sample that was more homogeneous with regard to mean differences. This is the standard problem of within-group versus between-group correlations that can lead to problems of inference (e.g., Yule, 1903; Simpson, 1951; Bickel, 1975).

Taking option (b) would involve performing separate factor analyses in each group and then estimating factor scores separately by group. Perhaps the most important advantage of this approach is that the factor analytic results would provide the best and closest fit to data within each sample. Further, factor analytic results would not be affected artifactually by group mean differences on the latent variable and hence on correlations among manifest variables. The disadvantages of this approach are notable. For example, the factor scoring weights would likely differ, at least to a degree, across samples, so any comparison across groups in mean levels or in correlations with other variables would have an “apples vs. oranges” aspect. Also, the typical approach to estimating factor scores using canned programs such as SPSS or SAS is first to standardize all manifest variables to *M* = 0.0 and *SD* = 1.0 and then to compute a differentially weighted sum of these standardized scores using factor scoring weights. The result is that factor scores for each group will have means of zero, erasing the ability to estimate and test mean differences across groups. To be sure, advanced approaches can be used to ensure that factors do not have means of zero in each group, but this would not avoid the problem of having different compositing weights in each sample.

**Sum scores**. Another option for scoring variables and then testing mean differences across groups is to use sum scores. The use of sum scores has several advantages: (a) it is a very simple option; (b) the same weights are used for all participants when computing the sum score; and (c) any group mean differences on manifest variables are not confounded in the process of determining the compositing weights. One disadvantage is that the use of sum scores requires the implicit assumption that a sum of the set of items into a single composite is justified, an assumption that could be investigated by conducting a factor analysis of the items in the sample. But then a decision would have to be made regarding how the factor analysis would be performed, and this would lead to the same problems as outline above. A second possible disadvantage is that sum scores might have somewhat lower levels of reliability than do estimated factor scores, but this is probably a relatively minor concern.

**Empirical example**. As one final set of analyses with the 14 Neuroticism items from the spi data set (see Table 7 of the manuscript), we used two methods to analyze differences between males and females to exemplify the differences in results from using estimated factor scores and sum scores. First, we performed factor analyses of the 14 items separately for females (*n* = 2348) and males (*n* = 1598) and estimated factor scores in each sample. Estimating factor scores in each sample led to mean values of 0.0 in each sample, thereby destroying the ability to investigate mean differences across samples. Even if some approach were used to ensure that factor means were not zero in each sample, the separate factor analyses in each group would lead to different compositing weights in each sample, leading to dubious interpretation of any mean difference across samples.

In contrast, using mean item scores, the mean difference between females (*M* = 3.94, *SD* = 0.97) and males (*M* = 3.43, *SD* = 1.03) was associated with a significant Cohen’s *d* of 0.51, 95% CI [0.45, 0.58], suggesting that females score, on average, about one-half *SD* higher than males on the Neuroticism scale. This difference in results for factor score estimates and unit-weighted sum (or mean) scores exemplifies the need to take great care in making cross-group comparisons and provides a clear example of one strength of unit-weighted composites.

Refer to standalone program entitled “prog_02.sex diffs on factor scores vs. sum score.R” for programming of the empirical example.

**Supplemental Tables**

Several supplemental tables are provided in the following pages. These tables, their labels, and contents are:

**Table S3**: This table is identical to Table 3 of the main manuscript, but is presented here to allow easy comparison with the following table. Table S3 contains indices of fit of alternative common factor models to the *unrounded* scaled scores (i.e., x06, x07, x09, x10, x12, x13). As discussed in the main manuscript, all three one-factor models (Models 1, 2, and 3) had very poor fit to the data. The three two-factor models had much improved levels of fit, and the two-factor congeneric model has optimal fit to the data, based on fit indices and the appropriateness of the specification of the model.

**Table S4**: Table S4 contains indices of fit of same set of alternative common factor models to the *rounded* scores (i.e., r06, r07, r09, r10, r12, r13). Comparison of fit indices in this table with those in the preceding table (Table S3) shows some differences in the fit of common factor models to rounded scores (Table S4) versus scaled scores (Table S3). In large measure, only relatively small differences will be seen, although some notable differences are present, especially the fit of the three one-factor models.

**Table S5**. This table contains common factor loadings and unique variances from three congeneric factor models fit to scaled scores (x06–x13), so is partially redundant with information in Table 4 of the main manuscript. The three models are: (a) a one-factor congeneric model, (b) a two-factor congeneric model, and (c) a bifactor congeneric model, with a single general factor and two orthogonal group factors.

**Table S6**. This table is formatted in the same way as the preceding table, Table S5, but contains results of congeneric CFA models fit to the *rounded* scores (r06–r13). Comparing results of Table S6 based on rounded scores against factor model estimates in Table S5 based on unrounded scaled scores will reveal modest differences in factor loadings, but larger and notable differences in error variances, which are consistently smaller in the analysis of unrounded scaled scores, but larger in analyses of rounded scores. This finding is consistent with our contention that rounding of scores on the six manifest variables reduced the precision of information in the scores, leading to larger proportions of error variance in the rounded scores.

**Table S7**. This table presents results of the estimation of coefficients α and ω_t for verbal and speed sum scores based on rounded scores, so results can be compared with results based on unrounded scaled scores in Table 5 of the manuscript. Comparison of results across these tables indicates that analyses of rounded scores led consistently to lower estimates of reliability than did analyses of (unrounded) scaled scores.

**Table S8**. This table contains statistics that allow the computation of composite reliability of verbal and speed sum scores based on rounded scores, so results can be compared with results based on unrounded scaled scores in Table 6 of the manuscript. Once again, analyses of rounded scores led to lower estimates of composite reliability, although the differences were relatively small.

**Table S9**. This table is formatted the same as Table 8 of the main manuscript, but contains parameter estimates, their *SE*s, and associated 95% CIs for alternative prediction models fit to the *rounded* scores (r06–r13). These prediction models involve the prediction of differences in performance on ability measures as a function of school. Comparison of values in this table with those in Table 8 will indicate whether basing analyses on rounded scaled scores (Table S9) versus unrounded scaled scores (Table 8) had any effect on results. Once again, in large measure, only relatively small differences will be noted.

**Table S10**. For persons wishing more standard (if old-style) reporting of statistical results (e.g., significance test values), Table S10 provides parameter estimates, their *SE*s, *z*-ratios, and associated *p*-values for all parameters in prediction models in manuscript Table 8, for prediction models fit to scaled scores.

**Table S11**. Table S11 is formatted the same as Table S10, but reports results for modeling of rounded scores. Thus, this table provides parameter estimates, their *SE*s, *z*-ratios, and associated *p*-values for all parameters in prediction models shown in Supplemental Table S10. Once again, relatively small differences will be seen.

Table S3

Fit of Six Alternative Factor Models to **Scaled** Scores (x06–x13)

| Model | No. of |  | Statistical fit | | |  | Practical fit | | | |
| --- | --- | --- | --- | --- | --- | --- | --- | --- | --- | --- |
| number | factors | Psychometric model | χ^2^ | *df* | Prob |  | RMSEA [CI] | CFI | TLI | SRMR |
| 1 | 1 | Essentially Parallel | 398.72 | 19 | <.0001 |  | .258 [.236, .280] | .430 | .550 | .254/195 |
| 2 | 1 | Essentially Tau Equivalent | 383.48 | 14 | <.0001 |  | .296 [.271, .322] | .446 | .406 | .251/.198 |
| 3 | 1 | Congeneric | 149.79 | 9 | <.0001 |  | .228 [.197, .261] | .789 | .648 | .130/.130 |
| 4 | 2 | Essentially Parallel | 40.62 | 16 | .0006 |  | .072 [.045, .099] | .963 | .965 | .104/.063 |
| 5 | 2 | Essentially Tau Equivalent | 29.10 | 12 | .004 |  | .069 [.037, .101] | .974 | .968 | .071/.049 |
| 6 | 2 | Congeneric | 14.35 | 8 | .073 |  | .051 [.000, .093] | .990 | .982 | .034/.034 |

*Note*: RMSEA [CI] = root mean square error of approximation and its 90% confidence interval, CFI = comparative fit index, TLI = Tucker–Lewis index, SRMR = standardized root mean square residual. ^a^ In the SRMR column, the value before the slash is from output based on analysis with Mplus, and the value after the slash is from output after analysis using lavaan package in R.

Table S4

Fit of Alternative Models to **Rounded** Scaled Scores (r06–r13) from McNeish and Wolf (2020)

| Model | No. of |  | Statistical fit | | |  | Practical fit | | | |
| --- | --- | --- | --- | --- | --- | --- | --- | --- | --- | --- |
| number | factors | Psychometric model | *χ*^2^ | *df* | Prob |  | RMSEA [CI] | CFI | TLI | SRMR^a^ |
| 1 | 1 | Essentially Parallel | 325.90 | 19 | <.0001 |  | .232 [.210, .254] | .446 | .562 | .237/.182 |
| 2 | 1 | Essentially Tau Equivalent | 310.60 | 14 | <.0001 |  | .265 [.240, .291] | .464 | .426 | .231/.184 |
| 3 | 1 | Congeneric | 115.37 | 9 | <.0001 |  | .198 [.167, .231] | .808 | .680 | .114/.114 |
| 4 | 2 | Essentially Parallel | 40.60 | 16 | .0006 |  | .071 [.044, .099] | .956 | .958 | .116/.066 |
| 5 | 2 | Essentially Tau Equivalent | 26.92 | 12 | .008 |  | .064 [.031, .097] | .973 | .966 | .063/.047 |
| 6 | 2 | Congeneric | 14.74 | 8 | .065 |  | .053 [.000, .095] | .988 | .977 | .031/.031 |

*Note*: RMSEA [CI] = root mean square error of approximation and its 90% confidence interval, CFI = comparative fit index, TLI = Tucker–Lewis index, SRMR = standardized root mean square residual. ^a^ In the SRMR column, the value before the slash is from output based on analysis with Mplus, and the value after the slash is from output after analysis using lavaan package in R.

Table S5

Raw Score Parameter Estimates for Three Congeneric CFA Models Fit to **Scaled** Scores (x06–x13)

|  |  | One-factor model | |  | Two-factor model | | |  | Bifactor model | | | |
| --- | --- | --- | --- | --- | --- | --- | --- | --- | --- | --- | --- | --- |
|  | Test |  | Error |  | Loading | | Error |  | Loading | | | Error |
| Variable | Description | Loading | variance |  | Verbal | Speed | variance |  | General | Verbal | Speed | variance |
| x06 | Paragraph comprehension | 0.98 | 0.38 |  | 0.98 | 0* | 0.38 |  | 0.50 | 0.85 | .0* | 0.38 |
| x07 | Sentence completion | 1.11 | 0.42 |  | 1.11 | 0* | 0.42 |  | 0.57 | 0.96 | .0* | 0.42 |
| x09 | Word meaning | 0.91 | 0.37 |  | 0.91 | 0* | 0.37 |  | 0.47 | 0.78 | .0* | 0.37 |
| x10 | Addition | 0.19 | 1.15 |  | 0* | 0.67 | 0.73 |  | 0.34 | .0* | 0.58 | 0.73 |
| x12 | Counting dots | 0.18 | 0.99 |  | 0* | 0.78 | 0.42 |  | 0.40 | .0* | 0.67 | 0.42 |
| x13 | Straight-curved capitals | 0.28 | 0.94 |  | 0* | 0.59 | 0.67 |  | 0.30 | .0* | 0.51 | 0.67 |
|  |  |  |  |  |  |  |  |  |  |  |  |  |
| Factor 1 | | 1.0* |  |  | 1.0* |  |  |  | 1.0* |  |  |  |
| Factor 2 | |  |  |  | 0.26 | 1.0* |  |  | .0* | 1.0* |  |  |
| Factor 3 | |  |  |  |  |  |  |  | .0* | .0* | 1.0* |  |

*Note*: Asterisked parameters were fixed to reported values to identify the solution.

Table S6

Raw Score Parameter Estimates for Three Congeneric CFA Models Fit to **Rounded** Scaled Scores (r06–r13)

|  |  | One-factor model | |  | Two-factor model | | |  | Bifactor model | | | |
| --- | --- | --- | --- | --- | --- | --- | --- | --- | --- | --- | --- | --- |
|  | Test | Factor | Error |  | Factor | | Error |  | Factor | | | Error |
| Variable | Description | Loading | variance |  | Verbal | Speed | variance |  | General | Verbal | Speed | variance |
| r06 | Paragraph comprehension | 0.96 | 0.44 |  | 0.97 | 0* | 0.43 |  | 0.51 | 0.82 | .0* | 0.43 |
| r07 | Sentence completion | 1.12 | 0.50 |  | 1.23 | 0* | 0.50 |  | 0.59 | 0.96 | .0* | 0.50 |
| r09 | Word meaning | 0.89 | 0.47 |  | 0.90 | 0* | 0.47 |  | 0.47 | 0.76 | .0* | 0.47 |
| r10 | Addition | 0.20 | 1.28 |  | 0* | 0.66 | 0.88 |  | 0.35 | .0* | 0.56 | 0.88 |
| r12 | Counting dots | 0.19 | 1.02 |  | 0* | 0.73 | 0.52 |  | 0.38 | .0* | 0.62 | 0.52 |
| r13 | Straight-curved capitals | 0.28 | 1.08 |  | 0* | 0.60 | 0.80 |  | 0.32 | .0* | 0.51 | 0.80 |
|  |  |  |  |  |  |  |  |  |  |  |  |  |
| Factor 1 | | 1.0* |  |  | 1.0* |  |  |  | 1.0* |  |  |  |
| Factor 2 | |  |  |  | 0.28 | 1.0* |  |  | .0* | 1.0* |  |  |
| Factor 3 | |  |  |  |  |  |  |  | .0* | .0* | 1.0* |  |

*Note*: Asterisked parameters were fixed to reported values to identify the solution.

Table S7

Coefficients α and ω_t for Verbal and Speed Sum Scores based on **Rounded** Scores from Holzinger and Swineford (1939)

| Variable names |  | Covariance matrix | | |  | Factor model | |  | Reliability | |
| --- | --- | --- | --- | --- | --- | --- | --- | --- | --- | --- |
| Verbal tests |  | r06 | r07 | r09 |  | *λ* | *θ* |  | *α* | *ω_t* |
| r06 Paragraph Comp |  | 1.369 | 1.088 | 0.869 |  | .970 | .428 |  |  |  |
| r07 Sentence Comp |  | 1.088 | 1.763 | 1.005 |  | 1.122 | .504 |  |  |  |
| r09 Word Meaning |  | 0.869 | 1.005 | 1.278 |  | .896 | .475 |  | .860 | .864 |
|  |  |  |  |  |  |  |  |  |  |  |
| Speed tests |  | r10 | r12 | r13 |  | *λ* | *θ* |  | *α* | *ω_t* |
| r10 Addition |  | 1.320 | 0.502 | 0.369 |  | .650 | .898 |  |  |  |
| r12 Counting Dots |  | 0.502 | 1.061 | 0.438 |  | .772 | .465 |  |  |  |
| r13 S-C Caps |  | 0.369 | 0.438 | 1.161 |  | .568 | .839 |  | .637 | .643 |

*Note*: For variable names, Comp = Comprehension, S-C Caps = Straight-Curved Capitals. In the Factor model columns, λ = factor loading, and θ = error/unique variance.

Table S8

Reliabilities of Verbal and Speed Composite Sum Scores based on **Rounded** Scores (r06–r13)

| Variable names |  | Raw covariance matrix | | |  |  |  | Reduced covariance matrix | | |  | Composite |
| --- | --- | --- | --- | --- | --- | --- | --- | --- | --- | --- | --- | --- |
| Verbal tests |  | r06 | r07 | r09 |  | *r*_xx_ |  | r06 | r07 | r09 |  | Reliability |
| r06 Paragraph Comp |  | 1.369 | 1.088 | 0.869 |  | .700 |  | 0.958 | 1.088 | 0.869 |  |  |
| r07 Sentence Comp |  | 1.088 | 1.763 | 1.005 |  | .790 |  | 1.088 | 1.393 | 1.005 |  |  |
| r09 Word Meaning |  | 0.869 | 1.005 | 1.278 |  | .860 |  | 0.869 | 1.005 | 1.099 |  |  |
|  |  | Sum = 10.334 | | |  |  |  | Sum = 9.375 | | |  | .907 |
|  |  |  |  |  |  |  |  |  |  |  |  |  |
|  |  | Raw covariance matrix | | |  |  |  | Reduced covariance matrix | | |  |  |
| Speed tests |  | r10 | r12 | r13 |  | *r*_xx_ |  | r10 | r12 | r13 |  |  |
| r10 Addition |  | 1.320 | 0.502 | 0.369 |  | .955 |  | 1.261 | 0.502 | 0.369 |  |  |
| r12 Count Dots |  | 0.502 | 1.061 | 0.438 |  | .930 |  | 0.502 | 0.987 | 0.438 |  |  |
| r13 S-C Caps |  | 0.369 | 0.438 | 1.161 |  | .885 |  | 0.369 | 0.438 | 1.028 |  |  |
|  |  | Sum = 6.160 | | |  |  |  | Sum = 5.893 | | |  | .957 |

*Note*: For variable names, Comp= Comprehension; S-C Caps = Straight-Curved Capitals. *r*_xx_ = reliability, calculated as the average of reliability estimates for the Pasteur and Grant-White schools (Holzinger & Swineford, 1939). Reduced covariance matrix is identical to raw covariance matrix, except that error variance has been subtracted from each variance on the diagonal. Composite reliability = (sum of elements of reduced covariance matrix) / (sum of elements of raw covariance matrix).

Table S9

Results of Predicting Ability Outcomes from School Membership Using **Rounded** Scaled Scores (r06–r13)

|  |  |  |  |  |  |  | Standardized estimates | | | | |
| --- | --- | --- | --- | --- | --- | --- | --- | --- | --- | --- | --- |
| Estimator |  | Outcome |  | Raw score estimates | |  | Outcome variables | |  | Outcomes and predictor | |
| and method |  | variable |  | *B (SE)* | 95% CI |  | *d* (*SE*) | 95% CI |  | *β* (*SE*) | 95% CI |
| MLR estimation | | |  |  |  |  |  |  |  |  |  |
|  |  |  |  |  |  |  |  |  |  |  |  |
| Method 1 |  | General |  | 0.99 (0.51) | [‒0.02, 2.00] |  | 0.22 (0.12) | [‒0.00, 0.45] |  | 0.11 (0.06) | [‒0.00, 0.22] |
|  |  |  |  |  |  |  |  |  |  |  |  |
| Method 2 |  | Verbal |  | **1.68** (0.36) | [0.98, 2.38] |  | **0.52** (0.11) | [0.32, 0.73] |  | **0.26** (0.05) | [0.16, 0.36] |
|  |  | Speed |  | **‒0.69** (0.28) | [‒1.25, ‒0.13] |  | **‒0.28** (0.11) | [‒0.50, ‒0.06] |  | **‒0.14** (0.06) | [‒0.25, ‒0.03] |
|  |  |  |  |  |  |  |  |  |  |  |  |
| Method 3 |  | Verbal |  | **0.54** (0.12) | [0.31, 0.77] |  | **0.56** (0.11) | [0.34, 0.78] |  | **0.28** (0.06) | [0.17, 0.39] |
|  |  | Speed |  | ‒0.17 (0.09) | [‒0.35, 0.01] |  | ‒0.26 (0.14) | [‒0.53, 0.01] |  | ‒0.13 (0.07) | [‒0.27, 0.00] |
|  |  |  |  |  |  |  |  |  |  |  |  |
| Method 4 |  | Verbal |  | **0.54** (0.12) | [0.31, 0.77] |  | **0.56** (0.11) | [0.34, 0.78] |  | **0.28** (0.06) | [0.17, 0.39] |
|  |  | Speed |  | ‒0.25 (0.15) | [‒0.54, 0.04] |  | **‒0.34** (0.17) | [‒0.68, ‒0.01] |  | **‒0.17** (0.09) | [‒0.34, ‒0.00] |
|  |  |  |  |  |  |  |  |  |  |  |  |
| ML estimation | | |  |  |  |  |  |  |  |  |  |
|  |  |  |  |  |  |  |  |  |  |  |  |
| Method 1 |  | General |  | 0.99 (0.51) | [‒0.01, 1.99] |  | 0.22 (0.11) | [‒0.00, 0.44] |  | 0.11 (0.06) | [‒0.00, 0.22] |
|  |  |  |  |  |  |  |  |  |  |  |  |
| Method 2 |  | Verbal |  | **1.68** (0.36) | [0.98, 2.38] |  | **0.52** (0.11) | [0.32, 0.73] |  | **0.26** (0.05) | [0.16, 0.37] |
|  |  | Speed |  | **‒0.69** (0.28) | [‒1.24, ‒0.13] |  | **‒0.28** (0.11) | [‒0.50, ‒0.06] |  | **‒0.14** (0.06) | [‒0.25, ‒0.03] |
|  |  |  |  |  |  |  |  |  |  |  |  |
| Method 3 |  | Verbal |  | **0.54** (0.12) | [0.31, 0.77] |  | **0.56** (0.11) | [0.34, 0.78] |  | **0.28** (0.06) | [0.17, 0.39] |
|  |  | Speed |  | ‒0.17 (0.09) | [‒0.35, 0.01] |  | ‒0.26 (0.14) | [‒0.53, 0.01] |  | ‒0.13 (0.07) | [‒0.27, 0.00] |
|  |  |  |  |  |  |  |  |  |  |  |  |
| Method 4 |  | Verbal |  | **0.54** (0.12) | [0.31, 0.77] |  | **0.56** (0.11) | [0.34, 0.78] |  | **0.28** (0.06) | [0.17, 0.39] |
|  |  | Speed |  | **‒0.25** (0.11) | [‒0.46, ‒0.04] |  | **‒0.34** (0.14) | [‒0.62, ‒0.07] |  | **‒0.17** (0.07) | [‒0.31, ‒0.04] |

*Note*: Boldfaced coefficients had 95% CIs that did not include zero, so were significant at *p* < .05.

Table S10

Results of Predicting Ability Outcomes from School Membership Using **Scaled** Scores (x06–x13):

With *z* and *p* values instead of and 95% CIs

|  |  |  |  |  |  |  |  | Standardized estimates | | | | | | |
| --- | --- | --- | --- | --- | --- | --- | --- | --- | --- | --- | --- | --- | --- | --- |
| Estimator |  | Dependent |  | Raw score estimates | | |  | Latent vars. |  | | All | |  |  |
| and method |  | variable |  | *B (SE)* | *z* | *p* |  | *d* (*SE*) |  | | *β* (*SE*) | | *z* | *p* |
| MLR estimation | | |  |  |  |  |  |  |  | |  | |  |  |
|  |  |  |  |  |  |  |  |  |  | |  | |  |  |
| Method 1 |  | General |  | 1.08 (0.51) | 2.13 | .034 |  | 0.24 (0.12) |  | | 0.12 (0.06) | | 2.13 | .033 |
|  |  |  |  |  |  |  |  |  |  | |  | |  |  |
| Method 2 |  | Verbal |  | 1.76 (0.35) | 4.96 | <.001 |  | 0.55 (0.10) |  | | 0.28 (0.05) | | 5.27 | <.001 |
|  |  | Speed |  | −0.68 (0.28) | −2.42 | .016 |  | −0.28 (0.11) |  | | −0.14 (0.06) | | −2.47 | .014 |
|  |  |  |  |  |  |  |  |  |  | |  | |  |  |
| Method 3 |  | Verbal |  | 0.58 (0.12) | 4.89 | <.001 |  | 0.59 (0.11) |  | | 0.29 (0.06) | | 5.29 | <.001 |
|  |  | Speed |  | −0.16 (0.09) | −1.80 | .072 |  | −0.24 (0.13) |  | | −0.12 (0.07) | | −1.85 | .065 |
|  |  |  |  |  |  |  |  |  |  | |  | |  |  |
| Method 4 |  | Verbal |  | 0.57 (0.12) | 4.89 | <.001 |  | 0.59 (0.11) |  | | 0.29 (0.06) | | 5.31 | <.001 |
|  |  | Speed |  | −0.22 (0.12) | −1.86 | .062 |  | −0.31 (0.15) |  | | −0.16 (0.07) | | −2.10 | .036 |
|  |  |  |  |  |  |  |  |  |  | |  | |  |  |
| ML estimation | | |  |  |  |  |  |  |  |  | |
|  |  |  |  |  |  |  |  |  |  | |  | |  |  |
| Method 1 |  | General |  | 1.08 (0.51) | 2.13 | .033 |  | 0.24 (0.11) |  | | 0.12 (0.06) | | 2.15 | .031 |
|  |  |  |  |  |  |  |  |  |  | |  | |  |  |
| Method 2 |  | Verbal |  | 1.76 (0.36) | 4.96 | <.001 |  | 0.55 (0.11) |  | | 0.28 (0.05) | | 5.26 | <.001 |
|  |  | Speed |  | −0.68 (0.28) | −2.43 | .015 |  | −0.28 (0.11) |  | | −0.14 (0.06) | | −2.46 | .014 |
|  |  |  |  |  |  |  |  |  |  | |  | |  |  |
| Method 3 |  | Verbal |  | 0.58 (0.12) | 4.89 | <.001 |  | 0.59 (0.11) |  | | 0.29 (0.06) | | 5.29 | <.001 |
|  |  | Speed |  | −0.16 (0.09) | −1.79 | .073 |  | −0.24 (0.13) |  | | −0.12 (0.07) | | −1.85 | .065 |
|  |  |  |  |  |  |  |  |  |  | |  | |  |  |
| Method 4 |  | Verbal |  | 0.57 (0.12) | 4.90 | <.001 |  | 0.59 (0.11) |  | | 0.29 (0.06) | | 5.30 | <.001 |
|  |  | Speed |  | −0.22 (0.10) | −2.25 | .025 |  | −0.31 (0.13) |  | | −0.16 (0.07) | | −2.32 | .023 |

Table S11

Results of Predicting Ability Outcomes from School Membership Using **Rounded** Scaled Scores (r06–r13):

With *z* and *p* values instead of and 95% CIs

|  |  |  |  |  |  |  |  | Standardized estimates | | | | | | |
| --- | --- | --- | --- | --- | --- | --- | --- | --- | --- | --- | --- | --- | --- | --- |
| Estimator |  | Dependent |  | Raw score estimates | | |  | Latent vars. |  | | All | |  |  |
| and method |  | variable |  | *B (SE)* | *z* | *p* |  | *d* (*SE*) |  | | *β* (*SE*) | | *z* | *p* |
| MLR estimation | | |  |  |  |  |  |  |  | |  | |  |  |
|  |  |  |  |  |  |  |  |  |  | |  | |  |  |
| Method 1 |  | General |  | 0.99 (0.51) | 1.93 | .054 |  | 0.22 (0.12) |  | | 0.11 (0.06) | | 1.93 | .054 |
|  |  |  |  |  |  |  |  |  |  | |  | |  |  |
| Method 2 |  | Verbal |  | 1.68 (0.36) | 4.70 | <.001 |  | 0.52 (0.11) |  | | 0.26 (0.05) | | 4.97 | <.001 |
|  |  | Speed |  | −0.69 (0.28) | −2.42 | .015 |  | −0.28 (0.11) |  | | −0.14 (0.06) | | −2.47 | .014 |
|  |  |  |  |  |  |  |  |  |  | |  | |  |  |
| Method 3 |  | Verbal |  | 0.54 (0.12) | 4.59 | <.001 |  | 0.56 (0.11) |  | | 0.28 (0.06) | | 4.94 | <.001 |
|  |  | Speed |  | −0.17 (0.09) | −1.83 | .067 |  | −0.26 (0.14) |  | | −0.13 (0.07) | | −1.90 | .058 |
|  |  |  |  |  |  |  |  |  |  | |  | |  |  |
| Method 4 |  | Verbal |  | 0.54 (0.12) | 4.59 | <.001 |  | 0.56 (0.11) |  | | 0.28 (0.06) | | 5.01 | <.001 |
|  |  | Speed |  | −0.25 (0.15) | −1.71 | .087 |  | −0.34 (0.17) |  | | −0.17 (0.09) | | −2.01 | .044 |
|  |  |  |  |  |  |  |  |  |  | |  | |  |  |
| ML estimation | | |  |  |  |  |  |  |  |  | |
|  |  |  |  |  |  |  |  |  |  | |  | |  |  |
| Method 1 |  | General |  | 0.99 (0.51) | 1.93 | .053 |  | 0.22 (0.11) |  | | 0.11 (0.06) | | 1.95 | .051 |
|  |  |  |  |  |  |  |  |  |  | |  | |  |  |
| Method 2 |  | Verbal |  | 1.68 (0.36) | 4.70 | <.001 |  | 0.52 (0.11) |  | | 0.26 (0.05) | | 4.95 | <.001 |
|  |  | Speed |  | −0.69 (0.28) | −2.44 | .015 |  | −0.28 (0.11) |  | | −0.14 (0.06) | | −2.47 | .013 |
|  |  |  |  |  |  |  |  |  |  | |  | |  |  |
| Method 3 |  | Verbal |  | 0.54 (0.12) | 4.59 | <.001 |  | 0.56 (0.11) |  | | 0.28 (0.06) | | 4.94 | <.001 |
|  |  | Speed |  | −0.17 (0.09) | −1.83 | .067 |  | −0.26 (0.14) |  | | −0.13 (0.07) | | −1.90 | .058 |
|  |  |  |  |  |  |  |  |  |  | |  | |  |  |
| Method 4 |  | Verbal |  | 0.54 (0.12) | 4.62 | <.001 |  | 0.56 (0.11) |  | | 0.28 (0.06) | | 4.98 | <.001 |
|  |  | Speed |  | −0.25 (0.11) | −2.37 | .018 |  | −0.34 (0.14) |  | | −0.17 (0.07) | | −2.47 | .014 |

**SAS Computer Program to Compute Coefficient *ω* for Bifactor Models**

An SAS computer script is provided for computing coefficient $\omega_{H}$(or reliability based only on the general factor) and coefficient $\omega_{T}$(or the total reliability, based on all factors), first from bifactor results based on rounded scores (shown in Table S6) and then from bifactor results based on unrounded scaled scores (shown in Table S5). The program script is followed by four pages of output from running the program. The output from this program shows that coefficient $\omega_{T}$ based on rounded scores is .818, whereas the coefficient $\omega_{T}$based on unrounded scaled scores is .846, underscoring our contention that rounding scores led to reduced precision and reduced reliability of the scores on the six tests from the HS battery.

The very low values for coefficient $\omega_{H}$, which is reliability based only on the general factor, of .345 and .343 for rounded and scaled scores, respectively, are yet another indicator that any “general” score across all six manifest variables—whether an estimated factor score or a sum score—has such low reliability that it is not a viable basis for analysis.

**SAS program to Calculate Omega Coefficients of Reliability**

options ls=**100** pagesize=**80** pageno=**1**;

**proc** **iml**;

*------------------------------------------------------------------------;

* Calculating OMEGA coefficients based on ROUNDED score bifactor model ;

*------------------------------------------------------------------------;

rg = { **.506** ,

**.589** ,

**.470** ,

**.346** ,

**.384** ,

**.315** };

rf = { **.821** **.000** ,

**.956** **.000** ,

**.762** **.000** ,

**.000** **.561** ,

**.000** **.624** ,

**.000** **.510** };

rm = { **1** **1** **1** **1** **1** **1** };

rt = { **.433** **.000** **.000** **.000** **.000** **.000** ,

**.000** **.496** **.000** **.000** **.000** **.000** ,

**.000** **.000** **.472** **.000** **.000** **.000** ,

**.000** **.000** **.000** **.881** **.000** **.000** ,

**.000** **.000** **.000** **.000** **.521** **.000** ,

**.000** **.000** **.000** **.000** **.000** **.798** };

r1 = rg * rg`;

r2 = rf * rf`;

rc = r1 + r2 + rt;

print 'General factor loadings, ROUNDED scores ' ,, rg ,,,

'Orthogonalized first-order loadings ' ,, rf ,,,

'Error variances/unique factor variances ' ,, rt ,,,

'Covariances recovered from general factor ' ,, r1 ,,,

'Covariances recovered from 1st order factors' ,, r2 ,,,

'Reproduced covariances among manifest vars ' ,, rc ,,, ;

rsumg = sum(rg);

rsumf = rm * rf;

rsumt = sum(rt);

rsumgsq = rsumg * rsumg;

rsumfsq = rsumf # rsumf;

print 'Sum of General factor loadings, ROUNDED scores ' ,, rsumg ,,,

'Sum of Orthogonalized first-order loadings ' ,, rsumf ,,,

'Sum of Error variances/unique factor variances ' ,, rsumt ,,,

'Squared sum of General factor loadings ' ,, rsumgsq ,,,

'Squared sums of first-order factor loadings ' ,, rsumfsq ,,, ;

rtotvar = sum (rsumgsq , rsumfsq , rsumt );

omegaH = rsumgsq / rtotvar ;

omegaT = ( sum (rsumgsq , rsumfsq) ) / rtotvar;

print 'Total variance represented by solution, ROUNDED scores' ,, rtotvar ,,,

'Omega Hierarchical, due to general factor alone ' ,, omegaH ,,,

'Omega Total, due to all common factors ' ,, OmegaT ,,, ;

*------------------------------------------------------------------------;

* Calculating OMEGA coefficients based on UNROUNDED score bifactor model ;

*------------------------------------------------------------------------;

xg = { **.503** ,

**.570** ,

**.465** ,

**.344** ,

**.396** ,

**.302** };

xf = { **.846** **.000** ,

**.958** **.000** ,

**.783** **.000** ,

**.000** **.579** ,

**.000** **.666** ,

**.000** **.509** };

xm = { **1** **1** **1** **1** **1** **1** };

xt = { **.382** **.000** **.000** **.000** **.000** **.000** ,

**.000** **.418** **.000** **.000** **.000** **.000** ,

**.000** **.000** **.367** **.000** **.000** **.000** ,

**.000** **.000** **.000** **.729** **.000** **.000** ,

**.000** **.000** **.000** **.000** **.422** **.000** ,

**.000** **.000** **.000** **.000** **.000** **.665** };

x1 = xg * xg`;

x2 = xf * xf`;

xc = x1 + x2 + xt;

print 'General factor loadings, UNROUNDED scores ' ,, xg ,,,

'Orthogonalized first-order loadings ' ,, xf ,,,

'Error variances/unique factor variances ' ,, xt ,,,

'Covariances recovered from general factor ' ,, x1 ,,,

'Covariances recovered from 1st order factors ' ,, x2 ,,,

'Reproduced covariances among manifest vars ' ,, xc ,,, ;

xsumg = sum(xg);

xsumf = xm * xf;

xsumt = sum(xt);

xsumgsq = xsumg * xsumg;

xsumfsq = xsumf # xsumf;

print 'Sum of General factor loadings, UNROUNDED scores ' ,, xsumg ,,,

'Sum of Orthogonalized first-order loadings ' ,, xsumf ,,,

'Sum of Error variances/unique factor variances ' ,, xsumt ,,,

'Squared sum of General factor loadings ' ,, xsumgsq ,,,

'Squared sums of first-order factor loadings ' ,, xsumfsq ,,, ;

xtotvar = sum (xsumgsq , xsumfsq , xsumt );

omegaH = xsumgsq / xtotvar ;

omegaT = ( sum (xsumgsq , xsumfsq) ) / xtotvar;

print 'Total variance represented by solution, UNROUNDED scores' ,, xtotvar ,,,

'Omega Hierarchical, due to general factor alone ' ,, omegaH ,,,

'Omega Total, due to all common factors ' ,, OmegaT ,,, ;

**run**;

**quit**;

**Output from SAS Program to Obtain Omega Estimates of Reliability**

The SAS System

General factor loadings, **ROUNDED** scores

rg

0.506

0.589

0.47

0.346

0.384

0.315

Orthogonalized first-order loadings

rf

0.821 0

0.956 0

0.762 0

0 0.561

0 0.624

0 0.51

Error variances/unique factor variances

rt

0.433 0 0 0 0 0

0 0.496 0 0 0 0

0 0 0.472 0 0 0

0 0 0 0.881 0 0

0 0 0 0 0.521 0

0 0 0 0 0 0.798

Covariances recovered from general factor

r1

0.256036 0.298034 0.23782 0.175076 0.194304 0.15939

0.298034 0.346921 0.27683 0.203794 0.226176 0.185535

0.23782 0.27683 0.2209 0.16262 0.18048 0.14805

0.175076 0.203794 0.16262 0.119716 0.132864 0.10899

0.194304 0.226176 0.18048 0.132864 0.147456 0.12096

0.15939 0.185535 0.14805 0.10899 0.12096 0.099225

Covariances recovered from 1st order factors

r2

0.674041 0.784876 0.625602 0 0 0

0.784876 0.913936 0.728472 0 0 0

0.625602 0.728472 0.580644 0 0 0

0 0 0 0.314721 0.350064 0.28611

0 0 0 0.350064 0.389376 0.31824

0 0 0 0.28611 0.31824 0.2601

Reproduced covariances among manifest vars

rc

1.363077 1.08291 0.863422 0.175076 0.194304 0.15939

1.08291 1.756857 1.005302 0.203794 0.226176 0.185535

0.863422 1.005302 1.273544 0.16262 0.18048 0.14805

0.175076 0.203794 0.16262 1.315437 0.482928 0.3951

0.194304 0.226176 0.18048 0.482928 1.057832 0.4392

0.15939 0.185535 0.14805 0.3951 0.4392 1.157325

Sum of General factor loadings, ROUNDED scores

rsumg

2.61

Sum of Orthogonalized first-order loadings

rsumf

2.539 1.695

Sum of Error variances/unique factor variances

rsumt

3.601

Squared sum of General factor loadings

rsumgsq

6.8121

Squared sums of first-order factor loadings

rsumfsq

6.446521 2.873025

Total variance represented by solution, **ROUNDED** scores

rtotvar

19.732646

Omega Hierarchical, due to general factor alone

omegaH

0.3452198

Omega Total, due to all common factors

omegaT

0.8175105

General factor loadings, **UNROUNDED** scores

xg

0.503

0.57

0.465

0.344

0.396

0.302

Orthogonalized first-order loadings

xf

0.846 0

0.958 0

0.783 0

0 0.579

0 0.666

0 0.509

Error variances/unique factor variances

xt

0.382 0 0 0 0 0

0 0.418 0 0 0 0

0 0 0.367 0 0 0

0 0 0 0.729 0 0

0 0 0 0 0.422 0

0 0 0 0 0 0.665

Covariances recovered from general factor

x1

0.253009 0.28671 0.233895 0.173032 0.199188 0.151906

0.28671 0.3249 0.26505 0.19608 0.22572 0.17214

0.233895 0.26505 0.216225 0.15996 0.18414 0.14043

0.173032 0.19608 0.15996 0.118336 0.136224 0.103888

0.199188 0.22572 0.18414 0.136224 0.156816 0.119592

0.151906 0.17214 0.14043 0.103888 0.119592 0.091204

Covariances recovered from 1st order factors

x2

0.715716 0.810468 0.662418 0 0 0

0.810468 0.917764 0.750114 0 0 0

0.662418 0.750114 0.613089 0 0 0

0 0 0 0.335241 0.385614 0.294711

0 0 0 0.385614 0.443556 0.338994

0 0 0 0.294711 0.338994 0.259081

Reproduced covariances among manifest vars

xc

1.350725 1.097178 0.896313 0.173032 0.199188 0.151906

1.097178 1.660664 1.015164 0.19608 0.22572 0.17214

0.896313 1.015164 1.196314 0.15996 0.18414 0.14043

0.173032 0.19608 0.15996 1.182577 0.521838 0.398599

0.199188 0.22572 0.18414 0.521838 1.022372 0.458586

0.151906 0.17214 0.14043 0.398599 0.458586 1.015285

Sum of General factor loadings, UNROUNDED scores

xsumg

2.58

Sum of Orthogonalized first-order loadings

xsumf

2.587 1.754

Sum of Error variances/unique factor variances

xsumt

2.983

Squared sum of General factor loadings

xsumgsq

6.6564

Squared sums of first-order factor loadings

xsumfsq

6.692569 3.076516

Total variance represented by solution, **UNROUNDED** scores

xtotvar

19.408485

Omega Hierarchical, due to general factor alone

omegaH

0.3429634

Omega Total, due to all common factors

omegaT

0.8463043

**Estimating Reliability of Sum Scores and Estimated Factor Scores**

**Nicewander (2020) approach**

Recently, Nicewander (2020) offered interesting insights into reliability of estimated factor scores and of sum scores. In a follow-up (W. Alan Nicewander, personal communication, August 2020), Nicewander provided simple matrix equations to estimate several important quantities. One common representation of reliability of a score is that it is the square of the correlation of that score with a true score. As a result, the reliability of an estimated factor score is the square of the correlation of the estimated factor score with the true factor score, and the reliability of a sum score is the square of the correlation of the sum score with the true factor score.

To estimate these quantities, here are some definitions:

$\mathbf{1}$ A (*p* x 1) vector of ones, used for summing

$\mathbf{x}$ A (*p* x 1) vector of deviation scores on *p* indicators

$\mathbf{1}'\mathbf{x}$ The sum score of an individual

$\mathbf{C}_{xx}$ Covariance matrix of *p* indicators (e.g., items), a (*p* x *p*) matrix

$\mathbf{\lambda}$ A (*p* x 1) matrix (or vector) of factor loadings

$f$ True common factor scores

$\hat{f}$ Estimated common factor scores, so$\hat{f}=\mathbf{\lambda}'\mathbf{C}_{xx}^{-1}\mathbf{x}$, or regression estimate scores

$\rho$ Symbol for population correlation

**Correlation of Estimated Factor Scores ($\hat{f}$) with True Factor Scores ($f$)**

$$\begin{matrix} \rho(\hat{f},f)=Cov(\hat{f},f)/[Var(\hat{f})]^{1/2} (f \text{is} \text{assumed} \text{to} \text{have} \text{unit} \text{variance)} \\ Cov(\hat{f},f)=E(\mathbf{\lambda}'\mathbf{C}_{xx}^{-1}\mathbf{x} f)=\mathbf{\lambda}'\mathbf{C}_{xx}^{-1}E(\mathbf{x} f)=\mathbf{\lambda}'\mathbf{C}_{xx}^{-1}\mathbf{\lambda} \\ Var(\hat{f})=E(\mathbf{\lambda}'\mathbf{C}_{xx}^{-1}\mathbf{x})(\mathbf{x}'\mathbf{C}_{xx}^{-1}\mathbf{\lambda})=\mathbf{\lambda}'\mathbf{C}_{xx}^{-1}\mathbf{\lambda} \\ \rho(\hat{f},f)= Cov(\hat{f},f)/[Var(\hat{f})]^{1/2}=\mathbf{\lambda}'\mathbf{C}_{xx}^{-1}\mathbf{\lambda}/[\mathbf{\lambda}'\mathbf{C}_{xx}^{-1}\mathbf{\lambda}]^{1/2}=[\mathbf{\lambda}'\mathbf{C}_{xx}^{-1}\mathbf{\lambda}]^{1/2} \end{matrix}$$

So, $\text{Reliability of }\hat{f}=\text{rel}(\hat{f})=\rho^{2}(\hat{f},f)=\mathbf{\lambda}'\mathbf{C}_{xx}^{-1}\mathbf{\lambda}$

**Correlation of Sum Scores ($\mathbf{1}'\mathbf{x}$) with True Factor Scores ($f$)**

$$\begin{matrix} \rho(\mathbf{1}'\mathbf{x},f)=Cov(\mathbf{1}'\mathbf{x},f)/[Var(\mathbf{1}'\mathbf{x})]^{1/2} \\ Cov(\mathbf{1}'\mathbf{x},f)=E(\mathbf{1}'\mathbf{x} f)=\mathbf{1}'E(\mathbf{x} f)=\mathbf{1}'\mathbf{\lambda}=\text{ sum of the factor loadings,} \lambda_{i} \\ Var\text{(}\mathbf{1}\text{'}\mathbf{x}\text{)} = E(\mathbf{1}'\mathbf{xx}'\mathbf{1}=\mathbf{1}'E(\mathbf{xx'})\mathbf{1}'=\mathbf{1}'\mathbf{C}_{xx}\mathbf{1}=\text{sum of the elements of }\mathbf{C}_{xx} \\ \rho(\mathbf{1}'\mathbf{x},f)=\mathbf{1}'\mathbf{\lambda}/[\mathbf{1}'\mathbf{C}_{xx}\mathbf{1}]^{1/2} \end{matrix}$$

So, $\text{Reliability of }\mathbf{1}'\mathbf{x}=\text{rel}(\mathbf{1}'\mathbf{x} )= \rho^{2}(\mathbf{1}'\mathbf{x},f)=\left(\mathbf{1}'\mathbf{x}\right)^{2}/\left[\mathbf{1}'\mathbf{C}_{xx}\mathbf{1}\right]$

The above is a matrix equation that is equivalent to McDonald’s coefficient omega.

**Correlation of Estimated Factor Scores ($\hat{f}$) with Sum Scores ($\mathbf{1}'\mathbf{x}$)**

The Nicewander approach also allows one to estimate the correlation of estimated factor scores and sum scores directly from the factor model estimates.

$$\begin{matrix} \text{Correlation of }\hat{f}\text{ and }\mathbf{1}'\mathbf{x}=\text{Cov}(\hat{f},\mathbf{1'x})/\left[\text{Var}(\hat{f})^{1/2}\text{Var}(\mathbf{1}\text{'}\mathbf{x})^{1/2}\right] \\ =E(\mathbf{1}'\mathbf{\lambda})/\left[(\mathbf{\lambda}'\mathbf{C}_{xx}^{-1}\mathbf{\lambda})^{1/2}(\mathbf{1}'\mathbf{C}_{xx}\mathbf{1})^{1/2}\right], \text{where} \mathbf{1'\lambda} \text{is} \text{Cov}(\hat{f},\mathbf{1}'\mathbf{x}) \end{matrix}$$

**Upshot**

The correlation of estimated factor scores and true factor scores leads directly to an estimate of the reliability of the estimated factor scores, which is not a lower bound estimate but a true estimate of reliability. The same goes for sum scores. With population data, these equations would provide ***the*** reliabilities of the estimated factor scores and sum scores. Of course, with sample data, the equations provide estimates of the reliabilities. In addition, it is nice to have a matrix equation for the correlation between estimated factor scores and sum scores.

**Important caveat**: The Nicewander matrix equations provide estimates of the reliability of estimated factor scores and of sum scores under the assumption that the factor model provides close fit to the covariances among indicators. If the factor model does not provide close fit to the observed covariances among indicators, some adjustments would be necessary.

**Cronbach (1951)/Rae (2007) Approach to Composite Reliability**

The key to estimating reliability is the separation of true score variance from total score variance. Cronbach (1951) provided inklings for how to estimate the reliability of a “lumpy” test, if one has access to the reliability of each component of the lumpy test. As one example, the three Verbal tests in the Holzinger and Swineford battery could be thought of as lumps in an overall test. The first lump is a homogeneous set of items for paragraph comprehension, the second lump is a homogeneous set of items requiring sentence comprehension, and the third lump is a homogeneous set of items involving word meaning. A similar characterization applies to the three Speed tests, as three lumps in an overall assessment of Speed.

Nicely, Holzinger and Swineford provided reliabilities for each of their tests. The sum of all elements in the (3×3) covariance matrix among verbal tests yields the total observed variance of the sum score across those tests. If one replaces each diagonal element in that matrix by the product of test reliability times test variance, one would obtain what we call a reduced covariance matrix, with estimated true score variances on the diagonal and observed covariances off the diagonal. The sum of elements in this reduced covariance matrix is an estimate of true score variance in the sum score across the tests.

The ratio of the sum of elements in the reduced covariance matrix divided by the sum of elements in the observed score matrix is an estimate of composite score reliability, which Cronbach called the reliability of a lumpy test.

**Following Pages with SAS PROC IML Programs and Output**

In the following pages, we provide three SAS PROC IML programs that implement the Nicewander equations and also the composite reliability for a lumpy test. These programs are:

Program #1: The ill-considered One-Factor Congeneric Test Model employed by McNeish and Wolf (2020), but using more precise **Scaled** scores from Holzinger and Swineford

Program #2: A One-Factor Congeneric Test Model for the Three Verbal **Scaled** (i.e., unrounded) Test Scores from Holzinger and Swineford

Program #3: A One-Factor Congeneric Test Model for the Three Speed **Scaled** (i.e., unrounded) Test Scores from Holzinger and Swineford

Programs for the Rounded scores used by McNeish and Wolf (2020) are minor variations on the above programs.

**References**

Cronbach, L. J. (1951). Coefficient alpha and the internal structure of tests. *Psychometrika*, *16*(3), 297-334.

Nicewander, W. A. (2020). A perspective on the mathematical and psychometric aspects of factor indeterminacy. *Multivariate Behavioral Research*, *55*(6), 825-838. <https://doi.org/10.1080/00273171.2019.1684872>

Rae, G. (2007). A note on using stratified alpha to estimate the composite reliability of a test composed of interrelated nonhomogeneous items. *Psychological Methods*, *12*(2), 177-184.

**PROGRAMS BASED ON NICEWANDER (2020)**

**AND ON CRONBACH (1951) AND RAE (2007)**

**SAS Program #1: The ill-considered One-Factor Congeneric Test Model employed by McNeish and Wolf (2020), but using more precise SCALED scores**

options ls=**96** pagesize=**100** pageno=**1**;

*------------------------------------------------------------------;

* First time through -- SCALED scores contra McNeish & Wolf ;

* ONE-FACTOR CONGENERIC FACTOR model results ;

* ALL SIX INDICATORS loading on one factor ;

* ;

* Using OBSERVED Cxx matrix in calculations ;

*------------------------------------------------------------------;

**data** hs3way;

infile 'c:\users\kwidaman\dropbox\mystuff\kw\mss\mss_in_prep_a_prep\widaman_revelle_data\nicewander\hs3way.txt' missover;

input case school grade female agemo

t06_para t07_sent t09_word t10_addi t12_coun t13_scca

x06_para x07_sent x09_word x10_addi x12_coun x13_scca

r06_para r07_sent r09_word r10_addi r12_coun r13_scca ;

**run**;

**proc** **means** data=hs3way;

**run**;

**quit**;

*------------------------------------------------------------------;

* data few == selects out the variables for analysis on this run ;

*------------------------------------------------------------------;

**data** few; set hs3way;

keep x06_para x07_sent x09_word x10_addi x12_coun x13_scca ;

**proc** **corr** data=few outp=few1 cov alpha;

**run**;

**proc** **print** data=few1;

**run**;

**quit**;

**data** fewcov; set few1; * select indicator covariance matrix ;

if _type_ eq 'COV';

**proc** **factor** data=few covariance outstat=few2 method=ml nfact=**1** rotate=none;

var x06_para x07_sent x09_word x10_addi x12_coun x13_scca ;

**run**;

**quit**;

**proc** **print** data=few2;

**run**;

**data** pattern; set few2; * select correlation factor pattern ;

if _type_ eq 'PATTERN';

**data** sds; set few2; * select indicator SDs ;

if _type_ eq 'STD';

**run**;

**proc** **iml**;

use fewcov;

read all var _num_ into cxx;

print cxx; * observed cxx matrix ;

use pattern;

read all var _num_ into rpat;

use sds;

read all var _num_ into sdsv;

lambda = (rpat # sdsv)`;

print lambda; * covariance metric factor loadings ;

unit = J(nrow(cxx), **1** , **1**); * column vector of unities ;

print unit;

*psychometric indices of true factor scores;

covfhatf = lambda` * inv(cxx) * lambda; * cov of fhat and f ;

varfhat = lambda` * inv(cxx) * lambda; * var of fhat ;

rhofhatf = covfhatf * inv(sqrt(varfhat)); * cor of fhat and f ;

relfhat = rhofhatf ** **2**; * square of the cor = ;

* rel of est fac scores ;

print 'HS DATA: ONE-FACTOR CONGENERIC MODEL ' ,,,

'loadings of indicators on factors ' ,, lambda ,,

'matrix of covariances among indicators ' ,, cxx ,,

'RELATIONS OF ESTIMATED FACTOR SCORES WITH TRUE FACTOR SCORES' ,,

'covariance of estimated and true factor scores' ,, covfhatf ,,

'variance of estimated factor scores ' ,, varfhat ,,

'correlation of estimated and true factor scores' ,, rhofhatf ,,

'RELIABILITY of ESTIMATED FACTOR SCORES ' ,, relfhat ,,,, ;

*psychometric indices of sum scores;

covsumf = unit` * lambda; * cov of sum score and f ;

varsum = unit` * cxx * unit; * var of sum score ;

rhosumf = covsumf * inv(sqrt(varsum)); * corr of sum score and f ;

relsum = rhosumf ** **2**; * square of the corr = ;

* rel of sum scores ;

print 'RELATIONS OF SUM SCORES WITH TRUE FACTOR SCORES' ,,,

'covariance of sum scores and true factor scores' ,, covsumf ,,

'variance of sum scores ' ,, varsum ,,

'correlation of sum scores and true factor scores' ,, rhosumf ,,

'RELIABILITY of SUM SCORES ' ,, relsum ,,,, ;

*relation between estimated factor scores and sum scores;

rhofhatsum = covsumf / ( sqrt(varfhat) * sqrt(varsum) );

print 'CORRELATION OF ESTIMATED FACTOR SCORES AND SUM SCORES' ,,

rhofhatsum ,,,, ;

*traditional indices of internal consistency reliability;

nitm = nrow(cxx); * number of items ;

itemvar = diag(cxx); * item variances ;

sumivar = unit` * itemvar * unit; * sum of item variances ;

alpha = (nitm / (nitm - **1**)) * (**1** - (sumivar * inv(varsum)));

truvar = (unit` * lambda) ** **2**; * true score variance ;

unqvar = diag(cxx - (lambda * lambda`)); * unique variances ;

sumuvar = unit` * unqvar * unit; * sum of unique variances ;

omega = truvar * inv(truvar + sumuvar);

print 'STANDARD INTERNAL CONSISTENCY RELIABILITY COEFFICIENTS' ,,,

'some preliminaries ' ,,

'number of items ' ,, nitm ,,

'item variances ' ,, itemvar ,,

'sum of item variances ' ,, sumivar ,,

'COEFFICIENT ALPHA ' ,, alpha ,,,,

'true score-related variance' ,, truvar ,,

'unique variances ' ,, unqvar ,,

'sum of unique variances ' ,, sumuvar ,,

'COEFFICIENT OMEGA ' ,, omega ;

averel = { **.700** **.000** **.000** **.000** **.000** **.000** ,

**.000** **.790** **.000** **.000** **.000** **.000** ,

**.000** **.000** **.860** **.000** **.000** **.000** ,

**.000** **.000** **.000** **.955** **.000** **.000** ,

**.000** **.000** **.000** **.000** **.930** **.000** ,

**.000** **.000** **.000** **.000** **.000** **.885** };

itemerr = itemvar - (itemvar * averel);

redcxx = cxx - itemerr;

sumcxx = sum(cxx);

sumred = sum(redcxx);

lumprel = sum(redcxx) / sum(cxx);

print 'RELIABILITY OF SUM OF LUMPY TEST: ALL SIX TESTS ' ,,,

'Raw covariances among the tests ' ,,

cxx (|format = **10.3**|) ,,

'Average reliabilities (across school) of SIX tests' ,,

averel (|format = **10.3**|) ,,

'Item error variances ' ,,

itemerr (|format = **10.3**|) ,,

'True score covariances among SIX tests ' ,,

redcxx (|format = **10.3**|) ,,

'Sum of raw covariances ' ,,

sumcxx (|format = **10.3**|) ,,

'Sum of elements in reduced covariance matrix ' ,,

sumred (|format = **10.3**|) ,,

'Reliability of a lumpy score across ALL SIX tests ' ,,

lumprel (|format = **10.3**|) ;

**run**;

**quit**;

**SAS Program #1: Selected Output**

analyses of unrounded SCALED HS test scores

unit

1

1

1

1

1

1

HS DATA: ONE-FACTOR CONGENERIC MODEL

loadings of indicators on factors

lambda

0.9852669

1.1133624

0.9114219

0.1930369

0.183431

0.275091

matrix of covariances among indicators

cxx

1.3551667 1.1014119 0.8985007 0.220475 0.126012 0.2441739

1.1014119 1.6653184 1.0179058 0.1434762 0.1812072 0.2962255

0.8985007 1.0179058 1.2003462 0.1445587 0.1659824 0.2367836

0.220475 0.1434762 0.1445587 1.1870833 0.5370294 0.3745415

0.126012 0.1812072 0.1659824 0.5370294 1.0253894 0.458841

0.2441739 0.2962255 0.2367836 0.3745415 0.458841 1.0183872

RELATIONS OF ESTIMATED FACTOR SCORES WITH TRUE FACTOR SCORES

RELIABILITY of ESTIMATED FACTOR SCORES

relfhat

0.8867559

RELATIONS OF SUM SCORES WITH TRUE FACTOR SCORES

RELIABILITY of SUM SCORES

relsum

0.6789947

CORRELATION OF ESTIMATED FACTOR SCORES AND SUM SCORES

rhofhatsum

0.8750465

STANDARD INTERNAL CONSISTENCY RELIABILITY COEFFICIENTS

COEFFICIENT ALPHA

alpha

0.7471459

COEFFICIENT OMEGA

omega

0.7587022

RELIABILITY OF SUM OF LUMPY TEST: ALL SIX TESTS

Raw covariances among the tests

cxx

1.355 1.101 0.899 0.220 0.126 0.244

1.101 1.665 1.018 0.143 0.181 0.296

0.899 1.018 1.200 0.145 0.166 0.237

0.220 0.143 0.145 1.187 0.537 0.375

0.126 0.181 0.166 0.537 1.025 0.459

0.244 0.296 0.237 0.375 0.459 1.018

Average reliabilities (across school) of ALL SIX tests

averel

0.700 0.000 0.000 0.000 0.000 0.000

0.000 0.790 0.000 0.000 0.000 0.000

0.000 0.000 0.860 0.000 0.000 0.000

0.000 0.000 0.000 0.955 0.000 0.000

0.000 0.000 0.000 0.000 0.930 0.000

0.000 0.000 0.000 0.000 0.000 0.885

Item error variances

itemerr

0.407 0.000 0.000 0.000 0.000 0.000

0.000 0.350 0.000 0.000 0.000 0.000

0.000 0.000 0.168 0.000 0.000 0.000

0.000 0.000 0.000 0.053 0.000 0.000

0.000 0.000 0.000 0.000 0.072 0.000

0.000 0.000 0.000 0.000 0.000 0.117

True score covariances among SLL SIX tests

redcxx

0.949 1.101 0.899 0.220 0.126 0.244

1.101 1.316 1.018 0.143 0.181 0.296

0.899 1.018 1.032 0.145 0.166 0.237

0.220 0.143 0.145 1.134 0.537 0.375

0.126 0.181 0.166 0.537 0.954 0.459

0.244 0.296 0.237 0.375 0.459 0.901

Sum of raw covariances

sumcxx

19.746

Sum of elements in reduced covariance matrix

sumred

18.579

Reliability of a lumpy test across ALL SIX tests a la Cronbach

lumprel

0.941

**SAS Program #2: Holzinger-Swineford VERBAL factor (3 SCALED indicators)**

options ls=**80** pagesize=**100** pageno=**1** nodate;

*------------------------------------------------------------------;

* here -- SCALED scores from Holzinger & Swineford ;

* ONE-FACTOR CONGENERIC FACTOR model results ;

* THREE VERBAL INDICATORS loading on VERBAL factor ;

* ;

* stay with DEVIATION SCORES / COVARIANCES all the way ;

*------------------------------------------------------------------;

**data** hs3way1;

infile 'c:\users\kwidaman\dropbox\mystuff\kw\mss\mss_in_prep_a_prep\widaman_revelle_data\nicewander\hs3way.txt' missover;

input case school grade female agemo

t06_para t07_sent t09_word t10_addi t12_coun t13_scca

x06_para x07_sent x09_word x10_addi x12_coun x13_scca

r06_para r07_sent r09_word r10_addi r12_coun r13_scca ;

**run**;

**proc** **means** data=hs3way1;

**run**;

**quit**;

**data** hs3way1; set hs3way1; * setting up SCALED scores ;

d06_para = x06_para; * d is for deviation scores ;

d07_sent = x07_sent;

d09_word = x09_word;

d10_addi = x10_addi;

d12_coun = x12_coun;

d13_scca = x13_scca;

z06_para = x06_para; * z is for z scores ;

z07_sent = x07_sent;

z09_word = x09_word;

z10_addi = x10_addi;

z12_coun = x12_coun;

z13_scca = x13_scca;

**proc** **standard** data=hs3way1 mean=**0** out=hs3way2;

var d06_para d07_sent d09_word d10_addi d12_coun d13_scca ;

**run**;

**proc** **standard** data=hs3way2 mean=**0** std=**1** out=hs3way;

var z06_para z07_sent z09_word z10_addi z12_coun z13_scca ;

**run**;

**proc** **means** data=hs3way;

title 'analyses of unrounded SCALED VERBAL HS test scores';

**run**;

**quit**;

**data** hs3way; set hs3way;

Vdsum = d06_para + d07_sent + d09_word;

Vzsum = z06_para + z07_sent + z09_word;

**proc** **corr** data=hs3way;

var Vdsum Vzsum;

**run**;

*------------------------------------------------------------------;

* data few == selects out the variables for analysis on this run ;

*------------------------------------------------------------------;

**data** few; set hs3way;

keep d06_para d07_sent d09_word ;

**proc** **corr** data=few alpha outp=few1 cov;

**run**;

**proc** **print** data=few1;

**run**;

**quit**;

**data** fewcov; set few1; * select indicator covariance matrix ;

if _type_ eq 'COV';

**proc** **factor** data=few covariance method=uls nfact=**1** rotate=none;

**run**;

**quit**;

**proc** **factor** data=few covariance outstat=few2 method=ml nfact=**1** rotate=none;

**run**;

**quit**;

**proc** **print** data=few2;

**run**;

**data** pattern; set few2; * select correlation factor pattern ;

if _type_ eq 'PATTERN';

**data** sds; set few2; * select indicator SDs ;

if _type_ eq 'STD';

**run**;

**proc** **iml**;

use fewcov;

read all var _num_ into cxx;

print cxx; * observed cxx matrix ;

use pattern;

read all var _num_ into rpat;

use sds;

read all var _num_ into sdsv;

lambda = (rpat # sdsv)`;

print lambda (|format = **10.5**|); * covariance metric factor loadings ;

unit = J(nrow(cxx), **1** , **1**); * column vector of unities ;

print unit;

unqvar = diag(cxx - (lambda * lambda`)); * unique variances ;

* relations of estimated factor scores with true factor scores;

* correlation of Method I est. factor scores with true factor scores ;

* fhatI = estimated factor scores using Method I, or Idealized variables;

rhofhatIf = sqrt (inv ( inv(lambda` * lambda) * lambda` * cxx * lambda * inv(lambda` * lambda) ) );

relfhatI = inv ( inv(lambda` * lambda) * lambda` * cxx * lambda * inv(lambda` * lambda) );

print 'correlation of Method I estimated and true factor scores' ,,

rhofhatIf (|format = **10.5**|) ,,

'RELIABILITY of METHOD I ESTIMATED FACTOR SCORES ' ,,

relfhatI (|format = **10.5**|) ;

* correlation of Method II est. factor scores with true factor scores ;

* fhatB = estimated factor scores using Method II, Bartlett's method ;

tem1 = ( inv(lambda` * inv(unqvar) * lambda) * lambda` * inv(unqvar) * lambda) ** **2**;

tem2 = ( inv(lambda` * inv(unqvar) * lambda) * lambda` * inv(unqvar) * cxx *

inv(unqvar) * lambda * inv(lambda` * inv(unqvar) * lambda) );

rhofhatBf = sqrt (tem1) / sqrt(tem2);

relfhatB = tem1 / tem2;

print 'correlation of Method II estimated and true factor scores' ,,

rhofhatBf (|format = **10.5**|) ,,

'RELIABILITY of METHOD II ESTIMATED FACTOR SCORES ' ,,

relfhatB (|format = **10.5**|) ;

* correlation of Method III est.factor scores with true factor scores ;

* fhatR = est. factor scores using Method III, Regression estimates ;

covfhatRf = lambda` * inv(cxx) * lambda; * covariance of fhat and f ;

varfhatR = lambda` * inv(cxx) * lambda; * variance of fhat ;

rhofhatRf = covfhatRf * inv(sqrt(varfhatR)); * correlation of fhat and f ;

relfhatR = rhofhatRf ** **2**; * square of correlation = ;

* rel. of est fac scores ;

print 'HS DATA: ONE-FACTOR CONGENERIC MODEL ' ,,

'loadings of indicators on factors ' ,,

lambda (|format = **10.5**|) ,,

'matrix of covariances among indicators ' ,,

cxx (|format = **10.5**|) ,,

'RELATIONS OF ESTIMATED FACTOR SCORES WITH TRUE FACTOR SCORES' ,,

'covariance of estimated and true factor scores' ,,

covfhatRf (|format = **10.5**|) ,,

'variance of estimated factor scores ' ,,

varfhatR (|format = **10.5**|) ,,

'correlation of estimated and true factor scores' ,,

rhofhatRf (|format = **10.5**|) ,,

'RELIABILITY of METHOD III ESTIMATED FACTOR SCORES ' ,,

relfhatR (|format = **10.5**|) ;

*psychometric indices of sum scores;

covsumf = unit` * lambda; * cov of sum score and f ;

varsum = unit` * cxx * unit; * variance of sum score ;

rhosumf = covsumf * inv(sqrt(varsum)); * corr of sum score and f ;

relsum = rhosumf ** **2**; * square of the corr = ;

* rel. of sum scores ;

print 'RELATIONS OF SUM SCORES WITH TRUE FACTOR SCORES' ,,

'covariance of sum scores and true factor scores' ,,

covsumf (|format = **10.5**|) ,,

'variance of sum scores ' ,,

varsum (|format = **10.5**|) ,,

'correlation of sum scores and true factor scores' ,,

rhosumf (|format = **10.5**|) ,,

'RELIABILITY of SUM SCORES ' ,,

relsum (|format = **10.5**|) ;

*relation between estimated factor scores and sum scores;

rhofhatsum = covsumf / ( sqrt(varfhatR) * sqrt(varsum) );

print 'CORRELATION OF ESTIMATED FACTOR SCORES AND SUM SCORES' ,,

rhofhatsum (|format = **10.5**|) ;

*traditional indices of internal consistency reliability;

nitm = nrow(cxx); * number of items ;

itemvar = diag(cxx); * item variances ;

sumivar = unit` * itemvar * unit; * sum of item variances ;

alpha = (nitm / (nitm - **1**)) * (**1** - (sumivar * inv(varsum)));

truvar = (unit` * lambda) ** **2**; * true score variance ;

unqvar = diag(cxx - (lambda * lambda`)); * unique variances ;

sumuvar = unit` * unqvar * unit; * sum of unique variances ;

omega = truvar * inv(truvar + sumuvar);

print 'STANDARD INTERNAL CONSISTENCY RELIABILITY COEFFICIENTS' ,,

'some preliminaries ' ,,

'number of items ' ,,

nitm ,,

'item variances ' ,,

itemvar (|format = **10.5**|) ,,

'sum of item variances ' ,,

sumivar (|format = **10.5**|) ,,

'COEFFICIENT ALPHA ' ,,

alpha (|format = **10.5**|) ,,

'true score-related variance' ,,

truvar (|format = **10.5**|) ,,

'unique variances ' ,,

unqvar (|format = **10.5**|) ,,

'sum of unique variances ' ,,

sumuvar (|format = **10.5**|) ,,

'COEFFICIENT OMEGA ' ,,

omega (|format = **10.5**|) ,, ;

averel = { **.700** **.000** **.000** ,

**.000** **.790** **.000** ,

**.000** **.000** **.860** };

itemerr = itemvar - (itemvar * averel);

redcxx = cxx - itemerr;

sumcxx = sum(cxx);

sumred = sum(redcxx);

lumprel = sum(redcxx) / sum(cxx);

print 'RELIABILITY OF SUM OF LUMPY TEST: VERBAL TESTS ' ,,,

'Raw covariances among the tests ' ,,

cxx (|format = **10.5**|) ,,

'Average reliabilities (across school) of VERBAL tests' ,,

averel (|format = **10.5**|) ,,

'Item error variances ' ,,

itemerr (|format = **10.5**|) ,,

'True score covariances among VERBAL tests ' ,,

redcxx (|format = **10.5**|) ,,

'Sum of raw covariances ' ,,

sumcxx (|format = **10.5**|) ,,

'Sum of elements in reduced covariance matrix ' ,,

sumred (|format = **10.5**|) ,,

'Reliability of a lumpy VERBAL test a la Cronbach ' ,,

lumprel (|format = **10.5**|) ;

print 'RELIABILITY OF SUM OF LUMPY TEST: VERBAL TESTS ' ,,,

'Raw covariances among the tests ' ,,

cxx (|format = **10.3**|) ,,

'Average reliabilities (across school) of VERBAL tests' ,,

averel (|format = **10.3**|) ,,

'Item error variances ' ,,

itemerr (|format = **10.3**|) ,,

'True score covariances among VERBAL tests ' ,,

redcxx (|format = **10.3**|) ,,

'Sum of raw covariances ' ,,

sumcxx (|format = **10.3**|) ,,

'Sum of elements in reduced covariance matrix ' ,,

sumred (|format = **10.3**|) ,,

'Reliability of a lumpy VERBAL test a la Cronbach ' ,,

lumprel (|format = **10.3**|) ;

**run**;

**quit**;

**SAS Program #2: Selected Output**

analyses of unrounded SCALED VERBAL HS test scores 11

cxx

1.3551667 1.1014119 0.8985007

1.1014119 1.6653184 1.0179058

0.8985007 1.0179058 1.2003462

lambda

0.98601

1.11704

0.91125

unit

1

1

1

correlation of Method I estimated and true factor scores

rhofhatIf

0.94113

RELIABILITY of METHOD I ESTIMATED FACTOR SCORES

relfhatI

0.88572

correlation of Method II estimated and true factor scores

rhofhatBf

0.94127

RELIABILITY of METHOD II ESTIMATED FACTOR SCORES

relfhatB

0.88600

HS DATA: ONE-FACTOR CONGENERIC MODEL

loadings of indicators on factors

lambda

0.98601

1.11704

0.91125

matrix of covariances among indicators

cxx

1.35517 1.10141 0.89850

1.10141 1.66532 1.01791

0.89850 1.01791 1.20035

RELATIONS OF ESTIMATED FACTOR SCORES WITH TRUE FACTOR SCORES

covariance of estimated and true factor scores

covfhatRf

0.88600

variance of estimated factor scores

varfhatR

0.88600

correlation of estimated and true factor scores

rhofhatRf

0.94127

RELIABILITY of METHOD III ESTIMATED FACTOR SCORES

relfhatR

0.88600

RELATIONS OF SUM SCORES WITH TRUE FACTOR SCORES

covariance of sum scores and true factor scores

analyses of unrounded SCALED VERBAL HS test scores 12

covsumf

3.01430

variance of sum scores

varsum

10.25647

correlation of sum scores and true factor scores

rhosumf

0.94121

RELIABILITY of SUM SCORES

relsum

0.88588

CORRELATION OF ESTIMATED FACTOR SCORES AND SUM SCORES

rhofhatsum

0.99994

RELIABILITY OF SUM OF LUMPY TEST: VERBAL TESTS

Raw covariances among the tests

analyses of unrounded SCALED VERBAL HS test scores 13

cxx

1.35517 1.10141 0.89850

1.10141 1.66532 1.01791

0.89850 1.01791 1.20035

Average reliabilities (across school) of VERBAL tests

averel

0.70000 0.00000 0.00000

0.00000 0.79000 0.00000

0.00000 0.00000 0.86000

Item error variances

itemerr

0.40655 0.00000 0.00000

0.00000 0.34972 0.00000

0.00000 0.00000 0.16805

True score covariances among VERBAL tests

redcxx

0.94862 1.10141 0.89850

1.10141 1.31560 1.01791

0.89850 1.01791 1.03230

Sum of raw covariances

sumcxx

10.25647

Sum of elements in reduced covariance matrix

sumred

9.33215

Reliability of a lumpy VERBAL test a la Cronbach

lumprel

0.90988

RELIABILITY OF SUM OF LUMPY TEST: VERBAL TESTS

Raw covariances among the tests

cxx

1.355 1.101 0.899

1.101 1.665 1.018

0.899 1.018 1.200

Average reliabilities (across school) of VERBAL tests

averel

0.700 0.000 0.000

0.000 0.790 0.000

0.000 0.000 0.860

Item error variances

itemerr

0.407 0.000 0.000

0.000 0.350 0.000

0.000 0.000 0.168

True score covariances among VERBAL tests

redcxx

0.949 1.101 0.899

1.101 1.316 1.018

0.899 1.018 1.032

Sum of raw covariances

sumcxx

10.256

Sum of elements in reduced covariance matrix

sumred

9.332

Reliability of a lumpy VERBAL test a la Cronbach

analyses of unrounded SCALED VERBAL HS test scores 14

lumprel

0.910

**SAS Program #3: Holzinger-Swineford SPEED factor (3 SCALED indicators)**

options ls=**80** pagesize=**100** pageno=**1** nodate;

*------------------------------------------------------------------;

* here -- SCALED scores from Holzinger & Swineford ;

* ONE-FACTOR CONGENERIC FACTOR model results ;

* THREE SPEED INDICATORS loading on SPEED factor ;

* ;

* stay with DEVIATION SCORES / COVARIANCES all the way ;

*------------------------------------------------------------------;

**data** hs3way1;

infile 'd:\dropbox\mystuff\kw\mss\mss_in_prep_a_prep\widaman_revelle_data\nicewander\hs3way.txt' missover;

input case school grade female agemo

t06_para t07_sent t09_word t10_addi t12_coun t13_scca

x06_para x07_sent x09_word x10_addi x12_coun x13_scca

r06_para r07_sent r09_word r10_addi r12_coun r13_scca ;

**run**;

**proc** **means** data=hs3way1;

**run**;

**quit**;

**data** hs3way1; set hs3way1; * setting up SCALED scores ;

d06_para = x06_para; * d is for deviation scores ;

d07_sent = x07_sent;

d09_word = x09_word;

d10_addi = x10_addi;

d12_coun = x12_coun;

d13_scca = x13_scca;

z06_para = x06_para; * z is for z scores ;

z07_sent = x07_sent;

z09_word = x09_word;

z10_addi = x10_addi;

z12_coun = x12_coun;

z13_scca = x13_scca;

**proc** **standard** data=hs3way1 mean=**0** out=hs3way2;

var d06_para d07_sent d09_word d10_addi d12_coun d13_scca ;

**run**;

**proc** **standard** data=hs3way2 mean=**0** std=**1** out=hs3way;

var z06_para z07_sent z09_word z10_addi z12_coun z13_scca ;

**run**;

**proc** **means** data=hs3way;

title 'analyses of unrounded SCALED SPEED HS test scores';

**run**;

**quit**;

**data** hs3way; set hs3way;

Sdsum = d10_addi + d12_coun + d13_scca;

Szsum = z10_addi + z12_coun + z13_scca;

**proc** **corr** data=hs3way;

var Sdsum Szsum;

**run**;

*------------------------------------------------------------------;

* data few == selects out the variables for analysis on this run ;

*------------------------------------------------------------------;

**data** few; set hs3way;

keep d10_addi d12_coun d13_scca ;

**proc** **corr** data=few alpha outp=few1 cov;

**run**;

**proc** **print** data=few1;

**run**;

**quit**;

**data** fewcov; set few1; * select indicator covariance matrix ;

if _type_ eq 'COV';

**proc** **factor** data=few covariance method=uls nfact=**1** rotate=none;

**run**;

**quit**;

**proc** **factor** data=few covariance outstat=few2 method=ml nfact=**1** rotate=none;

**run**;

**quit**;

**proc** **print** data=few2;

**run**;

**data** pattern; set few2; * select correlation factor pattern ;

if _type_ eq 'PATTERN';

**data** sds; set few2; * select indicator SDs ;

if _type_ eq 'STD';

**run**;

**proc** **iml**;

use fewcov;

read all var _num_ into cxx;

print cxx; * observed cxx matrix ;

use pattern;

read all var _num_ into rpat;

use sds;

read all var _num_ into sdsv;

lambda = (rpat # sdsv)`;

print lambda; * covariance metric factor loadings ;

unit = J(nrow(cxx), **1** , **1**); * column vector of unities ;

print unit;

unqvar = diag(cxx - (lambda * lambda`)); * unique variances ;

* relations of estimated factor scores with true factor scores;

* corr of Method I estimated factor scores with true factor scores ;

* fhatI = est factor scores using Method I, or Idealized variables ;

rhofhatIf = sqrt (inv ( inv(lambda` * lambda) * lambda` * cxx * lambda * inv(lambda` * lambda) ) );

relfhatI = inv ( inv(lambda` * lambda) * lambda` * cxx * lambda * inv(lambda` * lambda) );

print 'correlation of Method I estimated and true factor scores' ,,

rhofhatIf (|format = **10.5**|) ,,

'RELIABILITY of METHOD I ESTIMATED FACTOR SCORES ' ,,

relfhatI (|format = **10.5**|) ,, ;

* corr of Method II estimated factor scores with true factor scores ;

* fhatB = estimated factor scores using Method II, Bartlett's method ;

tem1 = ( inv(lambda` * inv(unqvar) * lambda) * lambda` * inv(unqvar) * lambda) ** **2**;

tem2 = ( inv(lambda` * inv(unqvar) * lambda) * lambda` * inv(unqvar) * cxx *

inv(unqvar) * lambda * inv(lambda` * inv(unqvar) * lambda) );

rhofhatBf = sqrt (tem1) / sqrt(tem2);

relfhatB = tem1 / tem2;

print 'correlation of Method II estimated and true factor scores' ,,

rhofhatBf (|format = **10.5**|) ,,

'RELIABILITY of METHOD II ESTIMATED FACTOR SCORES ' ,,

relfhatB (|format = **10.5**|) ;

* corr of Method III estimated factor scores with true factor scores ;

* fhatR = est factor scores using Method III, Regression estimates ;

covfhatRf = lambda` * inv(cxx) * lambda; * cov of fhat and f ;

varfhatR = lambda` * inv(cxx) * lambda; * var of fhat ;

rhofhatRf = covfhatRf * inv(sqrt(varfhatR)); * corr of fhat and f ;

relfhatR = rhofhatRf ** **2**; * square of the corr = ;

* rel of est fac scores ;

print 'HS DATA: ONE-FACTOR CONGENERIC MODEL ' ,,

'loadings of indicators on factors ' ,,

lambda (|format = **10.5**|) ,,

'matrix of covariances among indicators ' ,,

cxx (|format = **10.5**|) ,,

'RELATIONS OF ESTIMATED FACTOR SCORES WITH TRUE FACTOR SCORES' ,,

'covariance of estimated and true factor scores' ,,

covfhatRf (|format = **10.5**|) ,,

'variance of estimated factor scores ' ,,

varfhatR (|format = **10.5**|) ,,

'correlation of estimated and true factor scores' ,,

rhofhatRf (|format = **10.5**|) ,,

'RELIABILITY of METHOD III ESTIMATED FACTOR SCORES ' ,,

relfhatR (|format = **10.5**|) ;

*psychometric indices of sum scores;

covsumf = unit` * lambda; * cov of sum score and f ;

varsum = unit` * cxx * unit; * var of sum score ;

rhosumf = covsumf * inv(sqrt(varsum)); * corr of sum score and f ;

relsum = rhosumf ** **2**; * square of the corr = ;

* rel of sum scores ;

print 'RELATIONS OF SUM SCORES WITH TRUE FACTOR SCORES' ,,

'covariance of sum scores and true factor scores' ,,

covsumf (|format = **10.5**|) ,,

'variance of sum scores ' ,,

varsum (|format = **10.5**|) ,,

'correlation of sum scores and true factor scores' ,,

rhosumf (|format = **10.5**|) ,,

'RELIABILITY of SUM SCORES ' ,,

relsum (|format = **10.5**|) ;

*relation between estimated factor scores and sum scores;

rhofhatsum = covsumf / ( sqrt(varfhatR) * sqrt(varsum) );

print 'CORRELATION OF ESTIMATED FACTOR SCORES AND SUM SCORES' ,,

rhofhatsum (|format = **10.5**|) ;

*traditional indices of internal consistency reliability;

nitm = nrow(cxx); * number of items ;

itemvar = diag(cxx); * item variances ;

sumivar = unit` * itemvar * unit; * sum of item variances ;

alpha = (nitm / (nitm - **1**)) * (**1** - (sumivar * inv(varsum)));

truvar = (unit` * lambda) ** **2**; * true score variance ;

unqvar = diag(cxx - (lambda * lambda`)); * unique variances ;

sumuvar = unit` * unqvar * unit; * sum of unique variances ;

omega = truvar * inv(truvar + sumuvar);

print 'STANDARD INTERNAL CONSISTENCY RELIABILITY COEFFICIENTS' ,,

'some preliminaries ' ,,

'number of items ' ,,

nitm ,,

'item variances ' ,,

itemvar (|format = **10.5**|) ,,

'sum of item variances ' ,,

sumivar (|format = **10.5**|) ,,

'COEFFICIENT ALPHA ' ,,

alpha (|format = **10.5**|) ,,

'true score-related variance' ,,

truvar (|format = **10.5**|) ,,

'unique variances ' ,,

unqvar (|format = **10.5**|) ,,

'sum of unique variances ' ,,

sumuvar (|format = **10.5**|) ,,

'COEFFICIENT OMEGA ' ,,

omega (|format = **10.5**|) ;

averel = { **.955** **.000** **.000** ,

**.000** **.930** **.000** ,

**.000** **.000** **.885** };

itemerr = itemvar - (itemvar * averel);

redcxx = cxx - itemerr;

sumcxx = sum(cxx);

sumred = sum(redcxx);

lumprel = sum(redcxx) / sum(cxx);

print 'RELIABILITY OF SUM OF LUMPY TEST: SPEED TESTS ' ,,,

'Raw covariances among the tests ' ,,

cxx (|format = **10.5**|) ,,

'Average reliabilities (across school) of SPEED tests ' ,,

averel (|format = **10.5**|) ,,

'Item error variances ' ,,

itemerr (|format = **10.5**|) ,,

'True score covariances among SPEED tests ' ,,

redcxx (|format = **10.5**|) ,,

'Sum of raw covariances ' ,,

sumcxx (|format = **10.5**|) ,,

'Sum of elements in reduced covariance matrix ' ,,

sumred (|format = **10.5**|) ,,

'Reliability of a lumpy SPEED test a la Cronbach ' ,,

lumprel (|format = **10.5**|) ;

**run**;

**quit**;

**SAS Program #3: Selected Output**

analyses of unrounded SCALED SPEED HS test scores 2

cxx

1.1870833 0.5370294 0.3745415

0.5370294 1.0253894 0.458841

0.3745415 0.458841 1.0183872

lambda

0.6620913

0.8111107

0.5656946

unit

1

1

1

correlation of Method I estimated and true factor scores

rhofhatIf

0.84649

RELIABILITY of METHOD I ESTIMATED FACTOR SCORES

relfhatI

0.71655

correlation of Method II estimated and true factor scores

rhofhatBf

0.85975

RELIABILITY of METHOD II ESTIMATED FACTOR SCORES

relfhatB

0.73917

HS DATA: ONE-FACTOR CONGENERIC MODEL

loadings of indicators on factors

lambda

0.66209

0.81111

0.56569

matrix of covariances among indicators

cxx

1.18708 0.53703 0.37454

0.53703 1.02539 0.45884

0.37454 0.45884 1.01839

RELATIONS OF ESTIMATED FACTOR SCORES WITH TRUE FACTOR SCORES

covariance of estimated and true factor scores

covfhatRf

0.73917

variance of estimated factor scores

varfhatR

0.73917

correlation of estimated and true factor scores

rhofhatRf

0.85975

RELIABILITY of METHOD III ESTIMATED FACTOR SCORES

relfhatR

0.73917

RELATIONS OF SUM SCORES WITH TRUE FACTOR SCORES

covariance of sum scores and true factor scores

covsumf

2.03890

variance of sum scores

varsum

5.97168

correlation of sum scores and true factor scores

rhosumf

0.83435

RELIABILITY of SUM SCORES

relsum

0.69614

CORRELATION OF ESTIMATED FACTOR SCORES AND SUM SCORES

rhofhatsum

0.97045

RELIABILITY OF SUM OF LUMPY TEST: SPEED TESTS

Raw covariances among the tests

cxx

1.18708 0.53703 0.37454

0.53703 1.02539 0.45884

0.37454 0.45884 1.01839

analyses of unrounded SCALED SPEED HS test scores 13

Average reliabilities (across school) of SPEED tests

averel

0.95500 0.00000 0.00000

0.00000 0.93000 0.00000

0.00000 0.00000 0.88500

Item error variances

itemerr

0.05342 0.00000 0.00000

0.00000 0.07178 0.00000

0.00000 0.00000 0.11711

True score covariances among SPEED tests

redcxx

1.13366 0.53703 0.37454

0.53703 0.95361 0.45884

0.37454 0.45884 0.90127

Sum of raw covariances

sumcxx

5.97168

Sum of elements in reduced covariance matrix

sumred

5.72937

Reliability of a lumpy SPEED test a la Cronbach

lumprel

0.95942
